# Differential synaptic signaling responses in human cortical organoids after photon and proton irradiation

**DOI:** 10.1016/j.stemcr.2025.102777

**Published:** 2026-01-08

**Authors:** Yuting Jiang, Danieli Born Guerra, Daniëlle C. Voshart, Eline Hageman, Luiza Reali Nazario, Marc-Jan van Goethem, Rob P. Coppes, Lara Barazzuol

**Affiliations:** 1 Department of Radiation Oncology, University Medical Center Groningen, University of Groningen, Groningen, the Netherlands; 2 Department of Biomedical Sciences, University Medical Center Groningen, University of Groningen, Groningen, the Netherlands; 3 Department of Biomedical Sciences, PARTREC, University Medical Center Groningen, University of Groningen, Groningen, the Netherlands

**Keywords:** human cortical organoids, neural progenitor cells, paediatric neuro-oncology, radiotherapy, proton therapy

## Abstract

Brain tumors are the most common solid cancer in children, with radiotherapy being a primary treatment. Proton therapy, with its precise dose distribution, is increasingly being used in these patients to minimize damage to the developing brain. However, the biological effects of proton irradiation on the human brain remain unclear. To investigate this, human cortical organoids were exposed to conventional photons, plateau protons, and spread-out Bragg peak (SOBP) protons, followed by comparative transcriptomic profiling. While photons and protons induced similar transcriptional profiles characterized by apoptosis and downregulation of DNA replication, SOBP protons uniquely downregulated genes involved in brain development and synaptic signaling. Functional calcium imaging, cell deconvolution analysis, and immunostaining indicated that SOBP protons impaired neural network function, due to reduced synaptic density and loss in excitatory neuron progenitors. These findings underscore the distinct biological effects of SOBP protons and their potential impact on the developing brain.

## Highlights

• hCOs irradiated with photons and plateau protons elicit shared transcriptomic changes
• SOBP proton irradiation suppresses genes linked to neurodevelopment and synapses
• Neural network function is more strongly impaired after SOBP proton irradiation
• Reduced synapses and neural progenitors may underlie the effects of SOBP protons

## Graphical abstract

**Figure:**
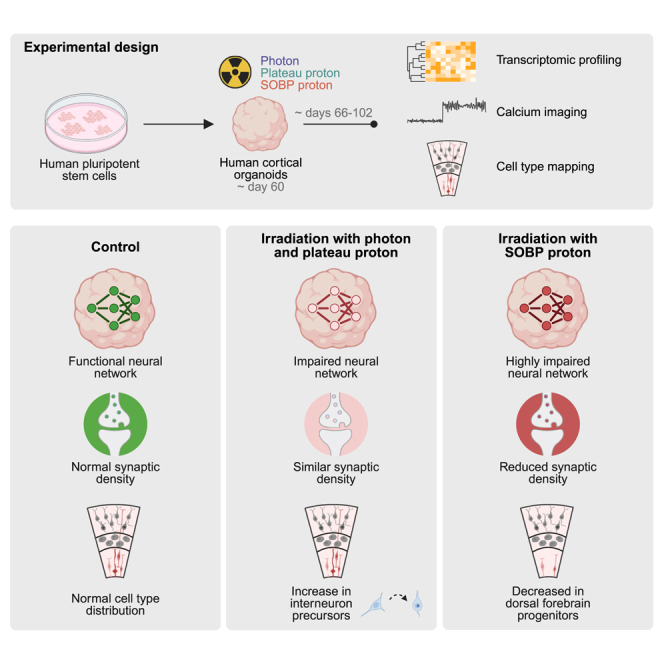

## Teaser

Pediatric brain tumor survivors often experience radiotherapy-induced cognitive decline. Jiang et al. found that compared to photons and plateau protons, SOBP protons more severely impair neural network function, potentially through disrupted synaptic density and loss of excitatory neural progenitors. These findings highlight the distinct biological effects of SOBP protons and their potential adverse impact on the developing brain.

## Introduction

Brain and central nervous system (CNS) tumors are the most common type of solid tumors in children and young adolescents (Siegel et al., 2023). Despite the heterogeneity of tumor types, radiotherapy remains a primary treatment modality in the management of many pediatric brain tumors. Compared to conventional photon-based radiotherapy, proton therapy offers advantages in dose distribution due to its unique physical properties, allowing more healthy tissue to be spared (Mohan and Grosshans, 2017). Unlike photons, protons lose only a small amount of energy in the entrance region, referred to as the plateau protons, and release all their remaining energy at the end of their path in a narrow peak known as the Bragg peak (Baumann et al., 2016; Gondi et al., 2016; Mohan and Grosshans, 2017; Padovani et al., 2012). By combining multiple Bragg peaks of varying energies and intensities, a spread-out Bragg peak (SOBP) can be created to cover the entire tumor volume (Daugherty et al., 2013; DeNunzio and Yock, 2020). The energy deposition drops dramatically after the peak, thus reducing radiation exposure to surrounding healthy tissues (DeNunzio and Yock, 2020). As proton therapy becomes more accessible, it has emerged as a favored treatment option for pediatric brain tumors, offering improved dose conformation and reduced radiation-induced adverse effects (Baumann et al., 2020; DeNunzio and Yock, 2020; Gondi et al., 2016).

With advances in treatment, the 5-year survival rate for pediatric brain tumor patients has increased to approximately 75% (Siegel et al., 2023). However, these survivors usually experience long-term cognitive impairment and IQ decline due to radiation exposure (Al Dahhan et al., 2022; Gondi et al., 2016; Zhang et al., 2018), which significantly impact their academic achievements, career prospects, and independence (Kunin-Batson et al., 2011; Mabbott et al., 2005). Currently, no effective treatments exist to prevent these adverse effects (Simó et al., 2024; Turnquist et al., 2020), highlighting the need to understand the cellular and molecular changes in the developing brain that drive radiation-induced cognitive dysfunction. Besides, most studies addressing these questions have focused on photon irradiation, with limited research on plateau or SOBP protons. Although proton therapy treatment planning assumes a relative biological effectiveness (RBE) of 1.1, it remains unclear whether photons and protons induce comparable biological effects on the brain, especially in the Bragg peak, where the linear energy transfer (LET) is much higher compared to the plateau region (Mohan and Grosshans, 2017; Sørensen et al., 2021). While most healthy tissue is exposed to plateau protons during proton therapy, tissue adjacent to the tumor may also receive SOBP protons, raising questions about whether SOBP exposure affects normal tissue differently than photons or plateau protons.

Animal models are widely used in preclinical research to study disease mechanisms; however, they have significant limitations in recapitulating the species-specific aspects of human brain development and tissue architecture (Eichmüller and Knoblich, 2022). While patient-derived materials, such as biopsies and postmortem brain tissues, can capture human-specific changes, they only provide limited snapshots of disease progression and are very difficult to obtain (Eichmüller and Knoblich, 2022). Human brain organoids, differentiated from human pluripotent stem cells (hPSCs), offer a promising model alternative. By applying exogenous cues that mimic endogenous patterning, brain region-specific organoids can be generated with cellular compositions and electrophysiological activities similar to those in the developing human brain (Trujillo et al., 2019; Velasco et al., 2019). These features have made brain organoids an increasingly valuable tool for modeling a range of neurodevelopmental conditions (Birey et al., 2022; Li et al., 2023; Di Lullo and Kriegstein, 2017; Samarasinghe et al., 2021; Sebastian et al., 2023).

In this study, we employ a human cortical organoid model to examine the effect of irradiation with photons, plateau protons, and SOBP protons on developing brain tissue. Our findings indicate that while photons and protons induce similar transcriptomic profiles characterized by increased apoptosis and cell-cycle arrest, SOBP protons uniquely downregulate genes involved in brain development and synaptic signaling. Further validation with live-cell calcium imaging recording, cell deconvolution analysis, and immunostaining suggests that reduced synaptic density, along with a decreased number of excitatory neural progenitors, likely contribute to the impaired neural network function observed after SOBP proton irradiation.

## Results

### RNA sequencing of human cortical organoids reveals similar transcriptional responses to photon and plateau proton irradiation

**Figure 1:**
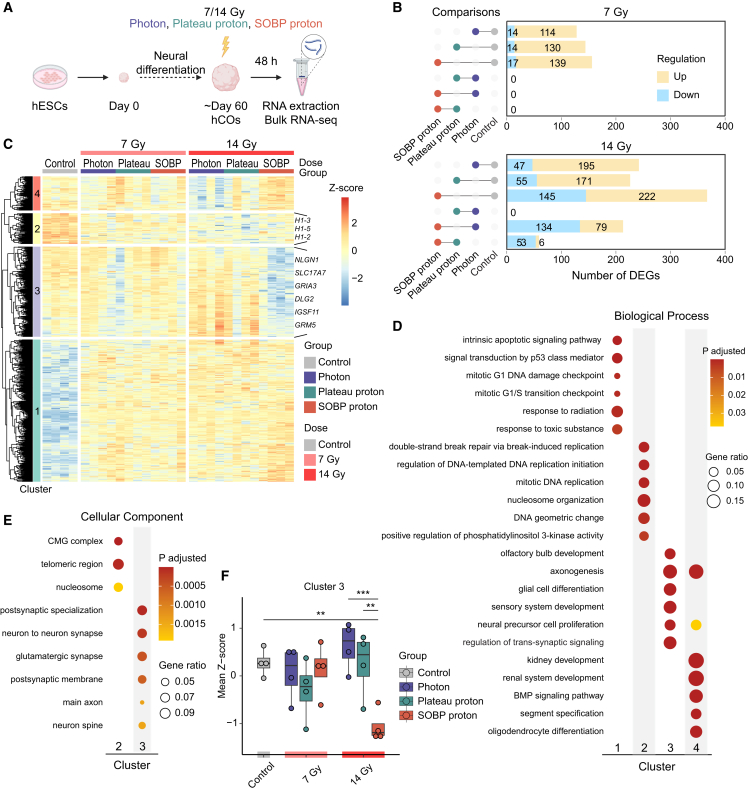
Comparative transcriptomic analysis of hCOs reveals unique effects of 14 Gy SOBP proton irradiation on brain development and synaptic signaling (A) Schematic diagram of the experiment. (B) Bar plot showing the number of DEGs across the different treatment groups among all the comparisons within the same radiation dose. (C) Heatmap with Manhattan distance-based hierarchical clustering analysis of all DEGs detected from all the comparisons in (B). 4 main clusters are identified. (D and E) Dot plots depicting the top six enriched GO terms for biological process (D) and cellular component (E) per cluster. (F) Boxplots showing the mean *Z* score of genes in cluster 3 for each condition. Boxes are drawn from first quartile to third quartile, with horizontal lines indicating the median. *n* = 4 pools per group, each pool contains 3–4 organoids. ^∗∗^*p* < 0.01 and ^∗∗∗^*p* < 0.001. Two-way ANOVA followed by Tukey’s multiple comparisons test was used for data shown in (F). See also Figure S1 and Table S1.

To investigate how different types of radiation affect the developing brain, we generated human cortical organoids (hCOs) from human embryonic stem cells (hESCs) following an established protocol (Sloan et al., 2018). At around 60 days of differentiation, the organoids were irradiated with photons, plateau protons, or SOBP protons at doses of 7 and 14 Gy. These doses correspond to fractionated schedules of 15.75 and 56 Gy, respectively, delivered in 2 Gy fractions, with the latter representing a clinically relevant dose (Fowler, 1989). We then assessed transcriptional changes 48 h post-irradiation using bulk RNA sequencing (RNA-seq) (Figure 1A).

To identify the biological processes affected by the different radiation types, we performed differential gene expression analysis across the different treatment groups. A dose-dependent increase in the number of differentially expressed genes (DEGs) was observed across the comparisons (Figure 1B). In line with our previous findings in adult rat brains (Voshart et al., 2024), photon and plateau proton irradiation induced similar transcriptional changes in hCOs, with no DEGs observed between these groups at either dose level (Figure 1B). While SOBP proton irradiation exhibited a transcriptional profile similar to the other groups irradiated with 7 Gy, it uniquely downregulated more genes at a higher dose of 14 Gy compared to photon or plateau proton irradiation (Figure 1B).

DEGs from all the comparisons were then divided into four clusters using Manhattan distance-based hierarchical clustering (Figure 1C) and annotated per cluster with Gene Ontology (GO) analysis (Figures 1D, 1E, and S1A). Clusters 1 and 2 comprised genes that were either upregulated or downregulated after irradiation in a dose-dependent manner, respectively, regardless of the radiation type (Figures 1C and S1B). Enriched biological processes indicated that the upregulated genes were associated with terms related to p53-mediated apoptotic pathways, while the downregulated genes were associated with terms related to DNA replication and nucleosome organization (Figure 1D). Cluster 4 included a small set of genes induced by 14 Gy SOBP proton irradiation while being downregulated by 14 Gy photon irradiation (Figures 1C and S1B), with GO terms related to development and BMP signaling pathway (Figure 1D).

### SOBP proton irradiation leads to a deregulation of brain development and synaptic signaling

In contrast, cluster 3 contained genes uniquely downregulated by 14 Gy SOBP proton irradiation but not by other radiation types (Figure 1F). These genes were associated with brain development processes, such as “olfactory bulb development,” “axonogenesis,” and “glial cell differentiation” (Figure 1D). Notably, about one-fourth of the genes in cluster 3 were synaptic genes as identified by SYNGO enrichment analysis (Table S1) (Koopmans et al., 2019), including *SLC17A7*, which encodes the pre-synaptic glutamate transporter VGLUT1, as well as *GRIA3* and *GRM5*, which encode different glutamate receptors (Figure 1C). GO analysis further confirmed disrupted synaptic function following 14 Gy SOBP proton irradiation, with enrichment in biological processes related to “regulation of *trans*-synaptic signaling” and cellular components such as “postsynaptic specialization” and “neuron to neuron synapse” (Figures 1D and 1E).

Overall, these results suggest that while photon and plateau proton irradiation induce similar transcriptional changes in hCOs characterized by a shared upregulation of apoptosis and downregulation of DNA replication, 14 Gy SOBP proton irradiation has a more distinct impact on brain development and synaptic signaling.

### SOBP proton irradiation more severely impairs neural network function

**Figure 2:**
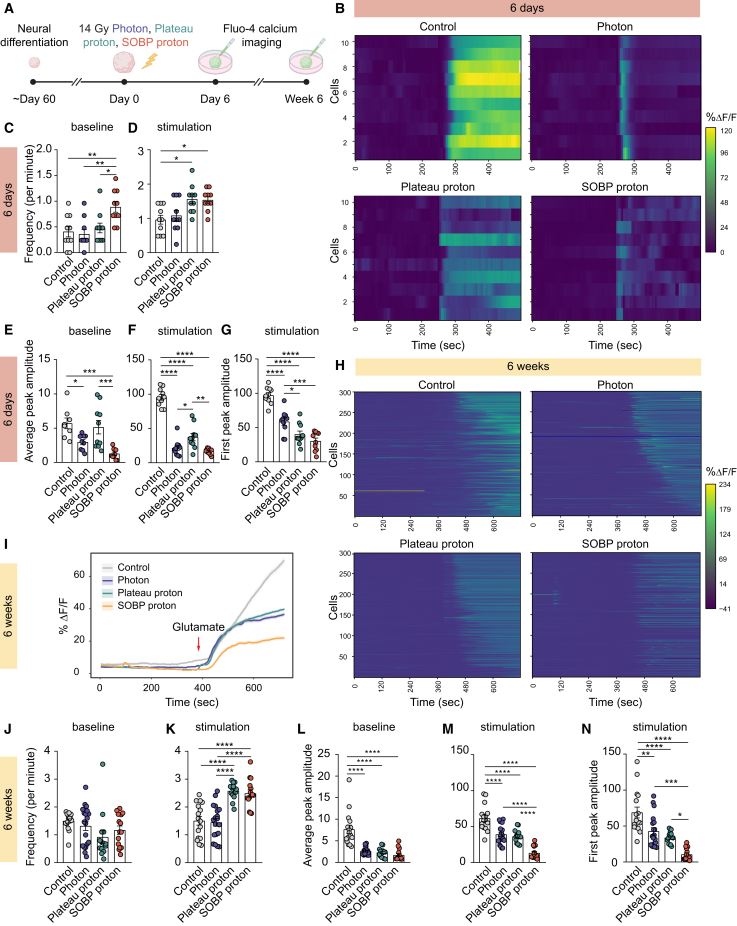
More pronounced impairment of neural activity after SOBP proton irradiation (A) Schematic diagram of the experiment. (B) Heatmap showing spontaneous and glutamate-induced calcium transients in 10 individual cells per group at 6 days post-irradiation. (C and D) Quantification of spontaneous (C) and glutamate-induced (D) calcium transient frequency (events per minute) in organoids at 6 days post-irradiation. (E and F) Quantification of average calcium transient peak amplitudes during baseline (E) and after glutamate stimulation (F) in organoids at 6 days post-irradiation. (G) Quantification of the first calcium transient peak amplitude following glutamate stimulation in organoids at 6 days post-irradiation. (H) Heatmap showing spontaneous and glutamate-induced calcium transients in 300 individual cells per group at 6 weeks post-irradiation. (I) Averaged calcium traces from 300 cells per group at 6 weeks post-irradiation before and after glutamate stimulation. Lines represent the mean, and shaded areas indicate the SEM. The red arrow indicates the addition of glutamate. (J and K) Quantification of spontaneous (J) and glutamate-induced (K) calcium transient frequency (events per minute) in organoids at 6 weeks post-irradiation. (L and M) Quantification of average calcium transient peak amplitudes during baseline (L) and after glutamate stimulation (M) in organoids at 6 weeks post-irradiation. (N) Quantification of the first calcium transient peak amplitude following glutamate stimulation in organoids at 6 weeks post-irradiation. *n* = 10 cells per group (C–G); *n* = 14–19 cells per group (J–N). Scatterplots show mean ± SEM (C–G and J–N). ^∗^*p* < 0.05, ^∗∗^*p* < 0.01, ^∗∗∗^*p* < 0.001, and ^∗∗∗∗^*p* < 0.0001. One-way ANOVA followed by Tukey’s multiple comparisons test was used for data shown in (C)–(G) and (J)–(N). See also Figure S2.

To validate whether the observed transcriptional changes led to functional alterations in neuronal network over time, we performed live-cell calcium imaging at 6 days and 6 weeks after 14 Gy photon, plateau proton, and SOBP proton irradiation (Figure 2A). hCOs were loaded with the calcium-sensitive dye Fluo-4, and calcium dynamics were recorded before and after the addition of glutamate, the major excitatory neurotransmitter in the brain. Changes in fluorescence intensity over time (ΔF/F(t)) for individual cells were plotted to assess neural activity (Figures 2B, 2H, and S2). At 6 days post-irradiation, photon exposure slightly reduced the amplitude of spontaneous calcium transients without altering their frequency, whereas plateau protons did not affect either frequency or amplitude (Figures 2C and 2E). SOBP proton irradiation, in contrast, resulted in more frequent but smaller transients compared to control and plateau proton-irradiated groups (Figures 2C and 2E). Upon glutamate stimulation, both plateau and SOBP proton irradiation increased the frequency of calcium transients (Figure 2D), while all radiation types reduced their peak amplitude (Figures 2F and 2G). At 6 weeks post-irradiation, the effect of SOBP proton irradiation on neuronal activity became more pronounced, as reflected in the averaged calcium transients (Figure 2I). Although no difference was observed in the frequency of spontaneous calcium transients across groups, all radiation types reduced their amplitude (Figures 2J and 2L). Interestingly, the increased frequency of calcium transients upon glutamate stimulation seen after plateau and SOBP proton irradiation at 6 days (Figure 2D) became more evident at 6 weeks (Figure 2K). Consistent with the early time point, all radiation types reduced the peak amplitude of glutamate-induced calcium transients at 6 weeks, with SOBP proton irradiation exerting the most pronounced effect (Figures 2M and 2N). This reduction in neuronal activity aligns with the transcriptional changes observed in synaptic genes, particularly those involved in glutamatergic signaling following SOBP proton irradiation (Figure 1E). In conclusion, these findings indicate that 14 Gy SOBP proton irradiation has a more profound effect on neural network activity compared to control group and irradiation with other radiation qualities.

### Reduced synaptic density may underlie impaired neural network function following SOBP proton irradiation

**Figure 3:**
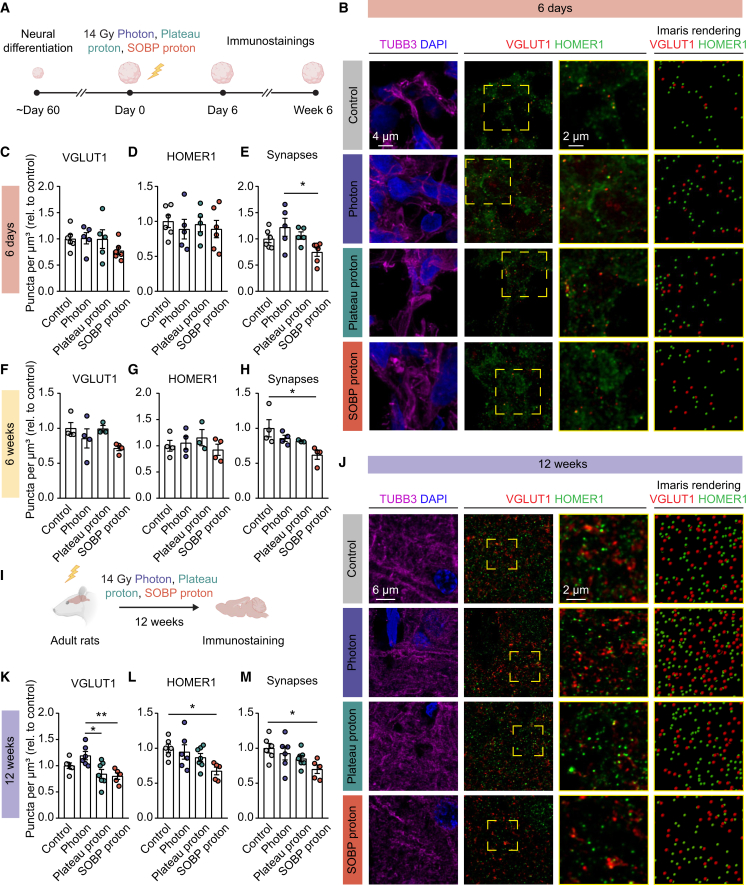
Changes in synaptic density after photon, plateau proton, and SOBP proton irradiation (A) Schematic diagram of the experiment. (B) Representative images of pre-synaptic protein VGLUT1 and post-synaptic protein HOMER1 in TUBB3^+^ neurons from 6-day post-irradiation organoids. The yellow dashed boxes are magnified in the images next to them. The final images show the 3D rendered VLGUT1 and HOMER1 puncta from insets using Imaris software. (C–E) Quantification of VGLUT1 puncta density (C), HOMER1 puncta density (D), and synaptic density (E) in 6-day post-irradiation organoids. (F–H) Quantification of VGLUT1 puncta density (F), HOMER1 puncta density (G), and synaptic density (H) in 6-week post-irradiation organoids. (I) Adult rats received 14 Gy photon, plateau proton, or SOBP proton irradiation and were sacrificed 12 weeks later. (J) Representative images of pre-synaptic protein VGLUT1 and post-synaptic protein HOMER1 in TUBB3^+^ neurons from the cortex of rat brains 12 weeks post-irradiation. The yellow dashed boxes are magnified in the images next to them. The final images show the 3D rendered VLGUT1 and HOMER1 puncta from insets using Imaris software. (K–M) Quantification of VGLUT1 puncta density (K), HOMER1 puncta density (L), and synaptic density (M) in the cortex of rat brains 12 weeks post-irradiation. *n* = 5–6 organoids per group (C–E). *n* = 3–4 organoids per group (F–H). *n* = 5–7 animals per group (K–M). Bar plots show mean ± SEM (C–E, F–H, and K–M). ^∗^*p* < 0.05, ^∗∗^*p* < 0.01. One-way ANOVA followed by Tukey’s multiple comparisons test was used for data shown in (C)–(E), (F)–(H), and (K)–(M).

Given the transcriptional and functional evidence of disrupted synaptic signaling following 14 Gy SOBP proton irradiation, we further validated these findings using immunostaining for VGLUT1, a presynaptic vesicular glutamate transporter, and HOMER1, a postsynaptic density scaffold protein, at both 6 days and 6 weeks post-irradiation (Figures 3A–3H). These proteins, both components of glutamatergic synapses, were quantified to assess synaptic density. No significant differences in VGLUT1 and HOMER1 density were observed between groups at either time point (Figures 3B–3D and 3F–3G). Although no reduction in synaptic density was detected at 6 days post-irradiation compared with the control (Figure 3E), SOBP proton irradiation significantly reduced synaptic density at 6 weeks, as indicated by decreased co-localization of VGLUT1 and HOMER1 puncta relative to the control group (Figure 3H). To determine whether SOBP proton irradiation induces a similar effect *in vivo*, we performed the same staining in adult rat brains 12 weeks after 14 Gy photon, plateau, or SOBP proton irradiation (Figures 3I and 3J). While radiation did not affect VGLUT1 density compared with control, SOBP protons significantly reduced HOMER1 expression and synaptic density in the cortex (Figures 3K–3M). Overall, these findings suggest that the reduction in synaptic number may contribute to the more pronounced neural network impairment seen after SOBP proton irradiation.

### SOBP proton irradiation reduces dorsal forebrain progenitor cells while increasing astrocyte proportion

**Figure 4:**
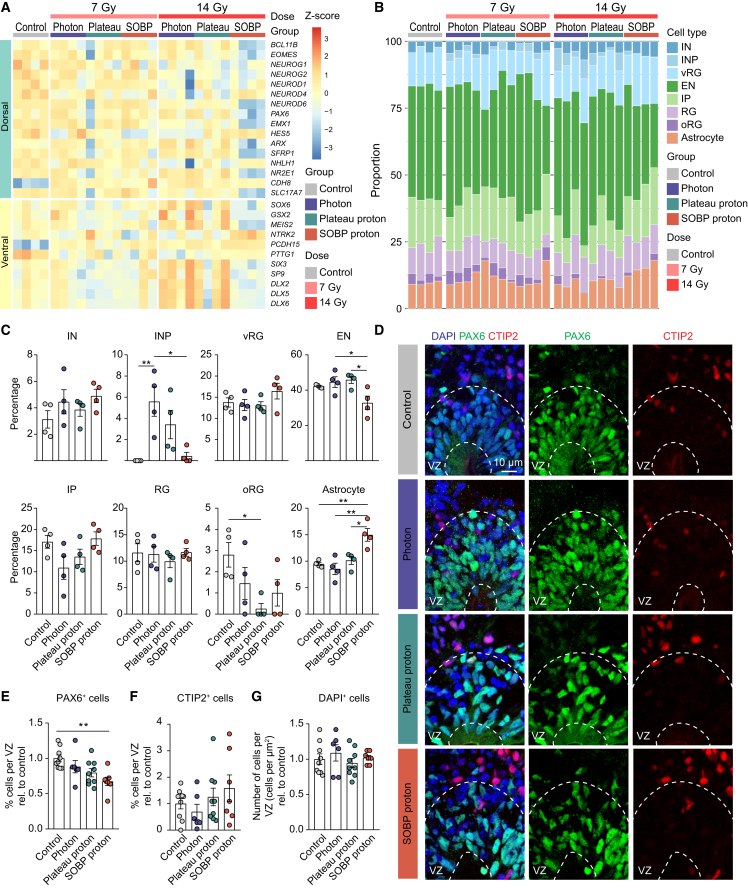
SOBP protons seem to decrease the proportion of neuronal progenitors while increasing astrocytes (A) Heatmap depicting expressions of differentially expressed regional brain identity markers. (B) Barplot showing the proportions of cell types in hCOs following cell deconvolution. (C) Individual bar plots illustrating changes in the percentage of each cell type following 14 Gy irradiation as shown in (B). (D) Representative images of PAX6 and CTIP2 with annotated ventricular zone (VZ). (E–G) Quantification of the percentage of PAX6^+^ cells (E), CTIP2^+^ cells (F), and the density of DAPI^+^ cells in VZ (G). *n* = 6–9 VZ per group. Bar plots show mean ± SEM (C, E, F, and G). ^∗^*p* < 0.05, ^∗∗^*p* < 0.01. One-way ANOVA followed by Tukey’s multiple comparisons test was used for data shown in (C), (E), (F), and (G). IN, interneuron; INP, interneuron precursor cell; vRG, ventral radial glial cell; EN, excitatory neuron; IP, intermediate progenitor cell; RG, radial glial cell; oRG, outer radial glial cell.

In the developing cerebral cortex, excitatory neurons arise from dorsal forebrain progenitors, while interneurons are mostly generated by progenitors in the ventral forebrain and later migrate to the cortical plate (Eichmüller and Knoblich, 2022). Since transcriptionally SOBP proton irradiation affects many brain development-related genes (Figure 1D), we examined whether different types of radiation also differentially affect regional brain identities. Irradiation with 14 Gy SOBP protons seems to decrease the expression of many dorsal forebrain markers at 48 h post-irradiation (Figure 4A). To gain further insight into how radiation affects individual cell types, we deconvolved our bulk RNA-seq data using CIBERSORTx (Newman et al., 2019). Photon irradiation significantly increases the proportion of interneuron precursor cells (INPs) compared to unexposed controls (Figures 4B and 4C). Additionally, 14 Gy SOBP irradiation appears to result in an increase in astrocytes (Figures 4B and 4C). We next investigated the impact of 14 Gy radiation at 6 days post-irradiation on the cellular composition of the ventricular zone (VZ), a region important for neurogenesis in the dorsal forebrain, with immunostaining for the dorsal forebrain neural progenitor cell marker PAX6 and deep-layer neuron marker CTIP2 (Figures 4D–4G). We found a significant decrease in PAX6^+^ cells within the VZ after SOBP proton irradiation (Figures 4D and 4E), with no significant differences in the percentage of CTIP2^+^ cells or total cell density (Figures 4F and 4G). Our findings suggest that SOBP proton irradiation leads to an increase in astrocytes and a decrease in dorsal forebrain progenitors within the VZ. These changes in cell composition may ultimately disrupt neural network function and brain development.

## Discussion

Clinical evidence, though mainly derived from small and non-randomized studies, suggests that proton therapy may improve cognitive outcomes compared to photon therapy, primarily owing to its more precise physical dose distribution (Mash et al., 2023; Sienna et al., 2024). Despite its widespread use as a preferred treatment modality for pediatric brain and CNS tumors, uncertainties persist regarding the biological and immunological differences between proton and photon radiation, as a number of studies have reported an increased incidence of imaging changes in patients after proton therapy (Gunther et al., 2015; Harrabi et al., 2022; Ludmir et al., 2019). Preclinical studies investigating the effects of photon and proton irradiation on normal brain tissue at the transcriptomic level are limited, with even fewer studies directly comparing plateau protons and SOBP protons. This gap of knowledge is particularly relevant, as normal tissue adjacent to the tumor may be exposed to SOBP protons, which are assumed to have higher RBE due to increased LET and, consequently, greater normal tissue damage than plateau protons received by most co-irradiated normal tissues.

A recent study demonstrated that 3 Gy photons and SOBP protons induce comparable early apoptotic responses in the neonatal mouse hippocampus and cerebellum (Giovannini et al., 2024). Consistent with this, our transcriptional data from the *in vitro* hCO model revealed that both radiation types induce apoptosis and cell-cycle arrest at similar levels in a dose-dependent manner. Moreover, our findings align with previous research showing that photon and plateau proton irradiation induce similar transcriptional profiles in the adult rat brain months after radiation exposure (Voshart et al., 2024). Similarly, our hCO model demonstrated early shared transcriptional changes between these two radiation types, suggesting a potential similarity in their effects and underscoring the utility of the hCO model for studying radiation effects on the developing brain.

Clinically, a fixed RBE of 1.1 is applied for proton therapy to account for its higher cell killing efficiency compared to photons (Paganetti et al., 2002; Sørensen et al., 2021). However, in practice, RBE is not constant and varies depending on factors such as tissue type, LET, fractionation dose, and biological endpoints (Sørensen et al., 2021; Zhou et al., 2024). Notably, an increase in RBE has been observed at the distal edge of the SOBP, a region where healthy tissues or critical organs are often located. This variability raises concerns, as clinical studies have reported radiation-induced imaging changes in this area (Bertolet et al., 2022; Engeseth et al., 2021; Winter et al., 2024). Cognitive dysfunction is a common long-term side effect of radiotherapy. Studies suggest that persistent radiation-induced changes in synaptic proteins, dendritic morphology, and spine density could contribute to cognitive decline (Parihar and Limoli, 2013; Parihar et al., 2015; Wu et al., 2022). While our study did not specifically investigate the distal edge of the SOBP, the pronounced effect of 14 Gy SOBP protons on brain development and synaptic signaling highlights the importance of refining proton therapy parameters, such as dose and energy distribution, to mitigate potential adverse effects such as (symptomatic) imaging changes and cognitive impairment in the developing brain.

Synapse formation during early brain development is essential for learning and memory. Disruptions in synapses or synapse-related genes and proteins have been associated with many neurodevelopmental, neurodegenerative, and psychiatric diseases (Dejanovic et al., 2024; Michetti et al., 2022; Wang et al., 2018). Alongside, recent studies have shown that brain tumor cells can hijack synaptic plasticity mechanisms leading to neuronal hyperexcitability, thus enhancing tumor growth and survival (Taylor et al., 2023). Our findings demonstrate that SOBP protons exert a more substantial impact on neuronal network function, raising the possibility that proton therapy may be more effective at disrupting neuron-to-glioma synapses, thus enhancing tumor control. Future investigations integrating hCO models with brain tumor organoids could help address this important question.

Our previous work also found that SOBP protons induce a greater expression of inflammation-related microglial priming genes than photons and plateau protons. However, the current hCO model lacks microglia, limiting our ability to investigate the interplay between microglia and neurons under different radiation conditions. Emerging techniques for generating brain organoids with integrated microglia could provide a more comprehensive understanding of radiation-induced biological changes in the developing brain (Sabogal-Guaqueta et al., 2024; Zhang et al., 2023).

Together, our data provide novel insights into the early responses of the human developing cortex to different types of radiation using hCOs as a model. While photon and plateau proton radiation lead to similar transcriptional profiles, SOBP proton radiation has a more pronounced impact on neural network function. Further investigations are needed to determine whether these effects translate into long-term functional consequences in the developing brain *in vivo*.

## Materials and methods

Due to word limitation, a detailed description of the methods is provided in the supplemental methods.

### hCO generation and irradiation

hCOs were generated following an established protocol (Sloan et al., 2018). Details are provided in the supplemental methods.

After approximately 60 days of differentiation, hCOs were irradiated with either 7 or 14 Gy of radiation. Photon irradiation was performed with Cesium-137 source (662 keV, dose rate 0.59 Gy/min) at the Department of Biomedical Sciences of UCMG. Proton irradiation, including both plateau protons (scattered beam, 150 MeV shoot-through, LET 0.5 keV/μm, dose rate 4 Gy/min) and SOBP protons (150 MeV, LET 3.1 keV/μm, dose rate 6 Gy/min), was conducted at the Particle Therapy Research Center (PARTREC) accelerator facility of the UMCG.

### Animal irradiation

Adult male Wistar (Hsd/Cpb:WU) rats were irradiated with 14 Gy photons or protons and sacrificed 12 weeks later. Details are provided in the supplemental methods.

### Bulk RNA-seq

RNA was isolated from hCOs 48 h after irradiation. For RNA isolation, library preparation, sequencing, and data analysis, see supplemental methods.

### Calcium imaging

Calcium activity in hCOs was visualized 6 days and 6 weeks after irradiation using the Fluo-4 Direct Calcium Kit (see supplemental methods).

### Immunofluorescence staining

hCOs were collected 6 days and 6 weeks post-irradiation for immunofluorescence staining. Details of the staining procedure, confocal imaging, and image analysis are provided in the supplemental methods.

### Statistical analysis

Statistical analysis was conducted using GraphPad Prism 8. Two-way analysis of variance (ANOVA) with Tukey’s multiple comparison test was applied to analyze statistical differences in mean *Z* score from RNA-seq data across groups within each radiation dose. For calcium imaging, immunofluorescence quantification, and cell proportion analysis from cell deconvolution data, one-way ANOVA with Tukey’s multiple comparison test was used. Statistical significance was defined as *p* < 0.05.

## Resource availability

### Lead contact

Further information and requests for resources reported in this paper should be directed to the lead contact, Lara Barazzuol (l.barazzuol@umcg.nl).

### Materials availability

This study did not generate any new reagents.

### Data and code availability

The accession number for the RNA-seq data reported in this paper is GEO: GSE311001. This paper does not generate any original code. Script and code can be requested from the lead contact.

## Acknowledgments

This work was supported by 10.13039/501100004622KWF Kankerbestrijding (project no. 11148 to L.B.), 10.13039/100009730Stand Up To Cancer (SU2C) and 10.13039/501100000289Cancer Research UK (CRUK) Pediatric Cancer New Discoveries Challenge Team Grant (project no. SU2C#RT6186 to L.B.), 10.13039/100016060Stichting De Cock-Hadders (project no. 2022-28 to D.C.V.), and 10.13039/501100004543China Scholarship Council (project no. 201906320080 to Y.J.). Some figures were created using BioRender.com.

## Author contributions

Conceptualization, L.B.; methodology, Y.J., D.B.G., D.C.V., and E.H.; investigation, Y.J., D.B.G., and E.H.; resources, D.C.V. and L.R.N.; writing – original draft, Y.J.; writing – review and editing, D.B.G., D.C.V., E.H., L.B., and R.P.C.; visualization, Y.J. and D.B.G.; supervision, L.B. and R.P.C.; funding acquisition, L.B. and D.C.V.

## Declaration of interests

The authors declare no competing interests.

## Declaration of generative AI and AI-assisted technologies in the writing process

During the preparation of this work, the first author used ChatGPT in order to improve readability. After using this tool, the authors reviewed and edited the content as needed and take full responsibility for the content of the publication.

## Supplemental information

Document S1. Figures S1 and S2 and supplemental methods

Table S1. SYNGO enrichment analysis of cluster 3 genes, related to Figure 1

Document S2. Article plus supplemental information
