# Supplementary material for: Differential synaptic signaling responses in human cortical organoids after photon and proton irradiation
Source: Stem Cell Reports. 2026 Jan 8;21(2):102777. doi: 10.1016/j.stemcr.2025.102777 (PMC12903084; doi:10.1016/j.stemcr.2025.102777)
Supplement: Document S1. Figures S1 and S2 and supplemental methods [file mmc1.pdf]

Stem Cell Reports, Volume *21*

Supplemental Information

Differential synaptic signaling responses in human cortical organoids after photon and proton irradiation

Yuting Jiang, Danieli Born Guerra, Daniëlle C. Voshart, Eline Hageman, Luiza Reali Nazario, Marc-Jan van Goethem, Rob P. Coppes, and Lara Barazzuol

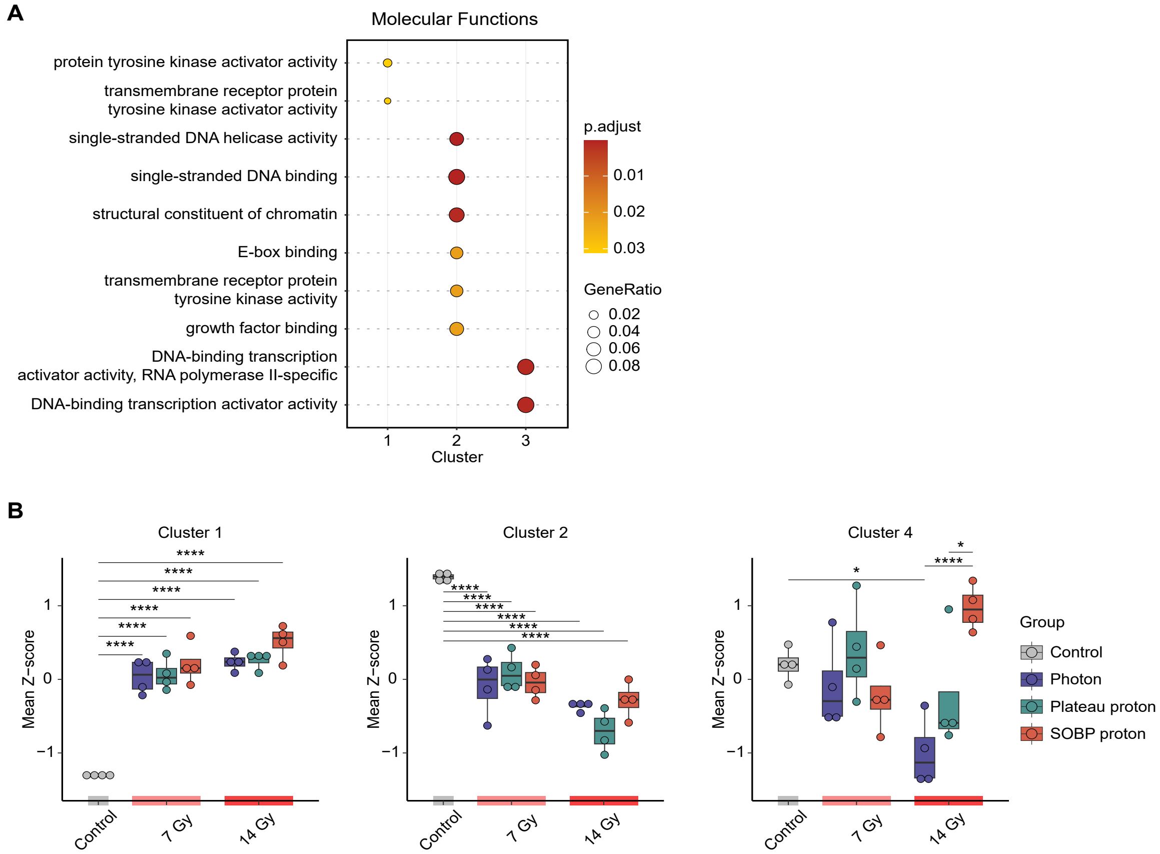

Figure S1. RNA-sequencing of hCOs after photon, plateau proton, and SOBP proton irradiation, related to Figure 1.

(A) Dot plot showing the top six enriched GO terms for molecular function in each cluster. (B) Boxplots showing the mean Z-score of genes in cluster 1, 2, and 4 for each condition. Boxes are drawn from first quartile to third quartile, with horizontal lines indicating the median. n = 4 pools per group, each pool contains 3-4 organoids. *p < 0.05 and ****p < 0.0001. Two-way ANOVA followed by Tukey's multiple comparisons test was used for data shown in (B).

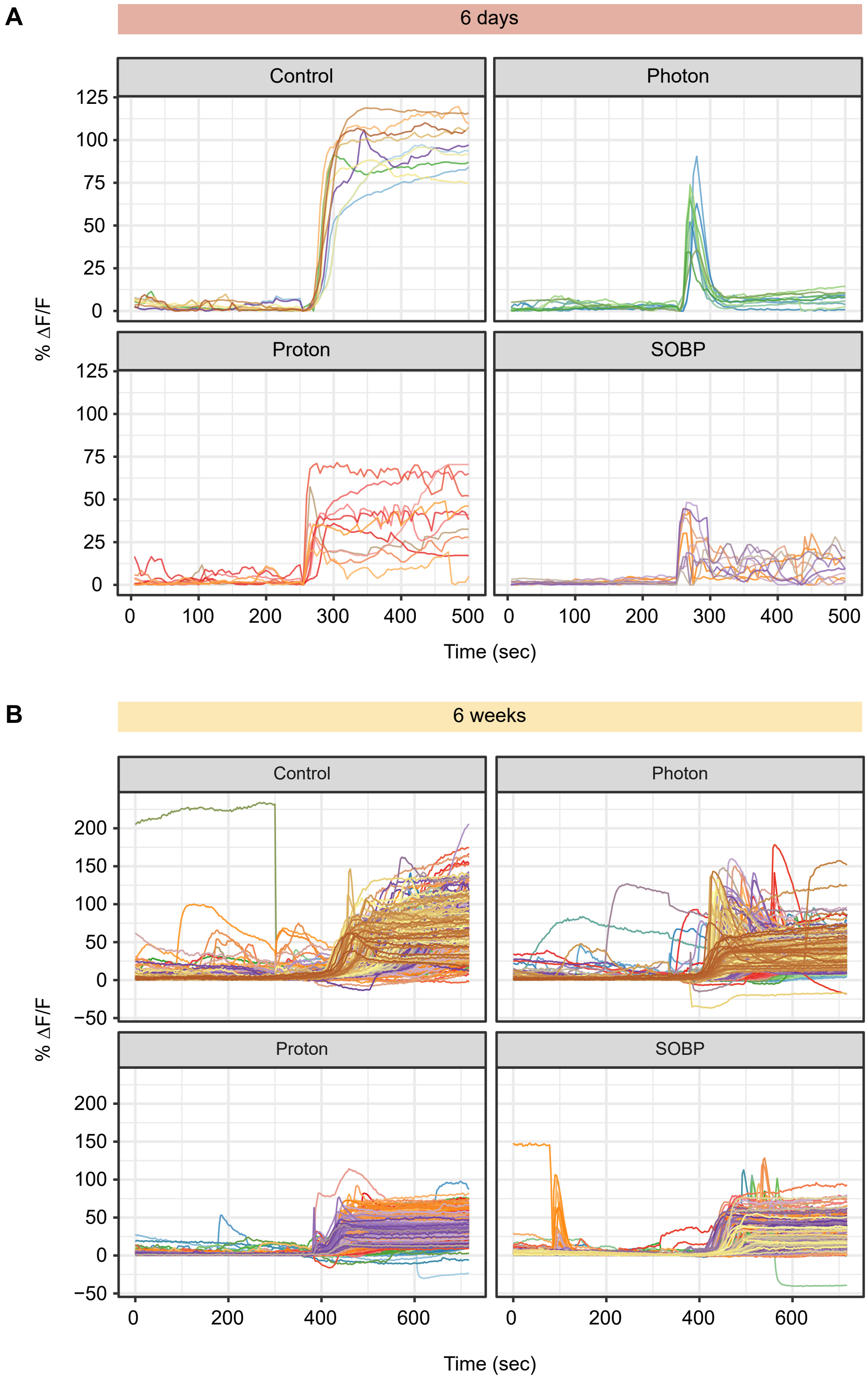

Figure S2. Individual spontaneous and glutamate-induced calcium traces at different time points after irradiation, related to Figure 2.

(A) Calcium traces from 10 cells per group at 6 days post-irradiation, with each line representing one cell. (B) Calcium traces from 300 cells per group at 6 weeks post-irradiation, with each line representing one cell.

Supplemental Methods

hCOs generation

HUES 9 (obtained from the Harvard Stem Cell Core Facility) hESCs were cultured on Matrigel (Corning, 734-1100) coated cell culture dishes using mTeSR1 medium (Stemcell Technologies, 85850). When HUES 9 cells were ready to be passaged, they were detached and dissociated into single cells using Accutase (Stemcell Technologies, 07920). The resulting cell suspension was counted and diluted in mTeSR1 medium containing ROCK inhibitor Y-27632 (1:100, Abcam, ab120129) before 3 million cells per well were seeded into AggreWell-800 plates (Stemcell Technologies, 34815). The plates were centrifuged at 100 g for 3 min to facilitate cell aggregation at the bottom of the microwells, which promotes uniform embryonic bodies (EB) formation. After 24h of incubation, EBs were transferred to ultra-low attachment T75 flasks (Merck, CLS3814) and cultured in neural induction medium (NIM), consisting of DMFM/F12 (Gibco, 11320-074), 20% knockout serum replacement (Gibco, 10828010), non-essential amino acids (1:100, Gibco, 11140050), Glutamax (Thermo Scientific, 35050038), 0.1 mM β-mercaptoethanol (Sigma, M3148), 1% penicillin-streptomycin (Gibco, 15140163), and supplemented with 5 μM dorsomorphin (Sigma, P5499-5MG) and 10 μM SB-431542 (Tocris, 1614). From day 6 onward, NIM was replaced daily with neural differentiation medium (NM) containing Neurobasal-A (Gibco, 10888022), 2% B-27 supplement without vitamin A (Gibco, 12587010), Glutamax (Thermo Scientific, 35050038), 1% penicillin-streptomycin (Gibco, 15140163), and supplemented with 20 ng/mL EGF (Sigma, E9644) and 20 ng/mL FGF-2 (PeproTech, 100-18B). Beginning on day 25, EGF and FGF-2 in NM were replaced by 20 ng/mL NT-3 (PeproTech, 450-03) and 20 ng/mL BDNF (PeproTech, 450-02). After day 43, the organoids were maintained in NM without any additional factors.

Animal irradiation

Adult male Wistar (Hsd/Cpb:WU) rats were kept under environmentally controlled conditions (temperature: 21°C, humidity: 55%) with a 12-hour light/dark cycle and ad libitum access to chow and water. The animals were housed in groups in open cages. All procedures were performed at the UMCG Central Animal Facility according to the guidelines from Directive 2010/63/EU of the European Parliament on the protection of animals used for scientific purposes. The experiments were approved by the Central Authority for Scientific Procedures on Animals (CCD) (license # AVD1050020184808) and the Animal Care and Use Committee of the University of Groningen. Rats were irradiated with 14 Gy photons or protons. Photon irradiations were conducted at the Central Animal Facility using an X-RAD 320 system (Precision X-Ray Inc., 200 kV, 1.559 Gy/min) with a specialized collimator for precise whole-brain targeting, as previously described (Voshart et al., 2024). Proton irradiations were performed at the Particle Therapy Research Center (PARTREC) accelerator facility, using either plateau protons (scattered beam, 150 MeV shoot-through, 15 Gy/min) or SOBP protons (4 Gy/min). Anesthesia was induced with 5% isoflurane and maintained at 1.5–2% during irradiation. Animals were sacrificed at 12 weeks post-irradiation by saline perfusion under dexmedetomidine-ketamine anesthesia. The brains were isolated and divided into two hemispheres, with one hemisphere fixed in 4% paraformaldehyde for 48 hours and subsequently embedded in paraffin for staining.

RNA isolation and RNA sequencing library preparation

RNA was isolated from hCOs 48 hours after irradiation using the RNeasy Lipid Tissue Mini Kit (Qiagen, 74804) following the manufacturer's instruction. Libraries were generated using the Lexogen QuantSeq 3' mRNA-seq Library Prep Kit (Lexogen, 015.96), with 200 ng of RNA used as input for each sample. All libraries were pooled in equimolar concentrations, and a 1.8 pM superpool with a 15% PhiX spike-in was sequenced on a NextSeq 500 using 75 bp single reads.

RNA sequencing data analysis

FASTQ files were aligned to the human genome (GRCh38) and pre-processed using the Quantseq Data Analysis Pipelines on the BlueBee Genomics Platform. Gene count matrices were used for the downstream analysis in R (v4.3.2). Genes with counts per million (CPM) greater than 2 in at least two samples were retained and normalized for differential gene expression analysis using edgeR (v4.0.14), with a threshold of 1.5 absolute fold change and an adjusted p-value < 0.05. GO analysis was conducted with clusterProfiler (v4.10.0) using the enrichGO function with a p-value and q-value cutoff of 0.05.

The CPM normalized gene expression matrix from this study was analyzed using the Docker version of CIBERSORTx to infer cell type compositions. Human single-cell brain organoid RNA-seq data containing control cells and cluster annotations were downloaded from

https://zenodo.org/record/7083558 (Li et al., 2023) and used to generate a custom signature matrix with the following arguments: --single_cell TRUE --replicates 200 --fraction 0.5 --verbose TRUE. Cell type fractions were then estimated using the following parameters: --perm 100 --rmbatchSmode TRUE --absolute FALSE.

Calcium imaging

Calcium activity in hCOs was visualized 6 days and 6 weeks after irradiation using the Fluo-4 Direct Calcium Kit (Invitrogen, F10471). Intact hCOs were placed in a 35 mm glass bottom plate containing NM without growth factors. Equal volumes of 2x Fluo-4 Direct calcium assay reagent were added to the medium. The hCOs were incubated at 37°C for 30 minutes, followed by 30 minutes at room temperature. The organoids were then transferred to the center of a crystal Petri dish for imaging. Fluorescence was recorded using a Leica DMI 8 microscope with a 40x objective for organoids at 6 days post-irradiation and a 10x objective for 6 weeks post-irradiation. Imaging was performed with an excitation wavelength of 494 nm and emission wavelength of 516 nm, capturing frames every 5 seconds for organoids at 6 days post-irradiation and every 3 seconds for 6 weeks post-irradiation. Basal calcium activity was recorded over 15 minutes. Stimulation experiments were performed by adding 4 µL of a 100 mM Glutamate stock solution directly to the Petri dish containing the organoid, and calcium responses were recorded for an additional 15 minutes.

Calcium imaging data were processed using ImageJ. Regions of interest (ROIs) representing individual cells were manually selected for organoids at 6 days post-irradiation and automatically segmented with the StarDist ImageJ plugin for organoids at 6 weeks post-irradiation. Mean gray values were measured for each frame. A total number of 10 cells were analyzed per condition at 6 days post-irradiation, and 300 cells were analyzed at 6 weeks post-irradiation. Relative changes in fluorescence ($\Delta F/F(t)$) were calculated using the formula:

$$\frac{\Delta F}{F(t)} = \frac{F_t - F_0}{F_0}$$

where ΔF would be the difference of fluorescence intensities between the current frame and F_0, with F_0 represents minimum gray values of the time series of each ROI. Calcium transients were identified when $\Delta F/F(t)$ exceeded a threshold of 2 median absolute deviations. The peak amplitude was measured from the median line to the maximum value of the peak. For calcium transient frequency and amplitude analysis, 10 cells per condition were analyzed at 6 days post-irradiation, and 20 randomly selected cells out of 300 per condition were analyzed at 6 weeks post-irradiation. Cells that did not respond to glutamate within the analyzed time frame were excluded, resulting in 14-19 cells quantified per group at 6 weeks post-irradiation.

Immunofluorescence staining

hCOs were collected 6 days post-irradiation and fixed an hour at room temperature in 4% PFA. After fixation, the organoids were transferred to 30% sucrose at 4 °C until they sank, and then embedded in OCT compound. Sections of 10 µm thickness were cut using a Leica Cryostat (Leica, CM1860). Cryosections were allowed to re-equilibrate at room temperature for 30 minutes, followed by a 10 minute wash in PBS to remove OCT. Antigen retrieval was performed using 10 mM sodium citrate buffer with 0.05% Tween-20 (pH 6.0). Sections were then blocked for 1 hour at room temperature in blocking solution containing 4% goat or donkey serum depending on the species of secondary antibodies, 1% BSA, and 0.1% Triton X-100 in PBS, and incubated overnight at 4 °C with the following primary antibodies diluted in blocking solution: chicken anti-Homer1 (Synaptic Systems, 160 006, 1:500, RRID: AB_2631222), guinea pig anti-VGLUT1 (Synaptic Systems, 135 304, 1:2000, RRID: AB_887878), rabbit anti-TUBB3 (Sigma, T2200, 1:500, RRID: AB_262133), rat anti-CTIP2 (Abcam, ab18465, 1:250, RRID: AB_2064130), rabbit anti-PAX6 (BioLegend, 901301, 1:100, RRID: AB_2565003). After primary antibody incubation, sections were washed and incubated for 1 hour at room temperature with appropriate secondary antibodies diluted in blocking solution: Alexa Fluor 488 goat anti-chicken (Invitrogen, A-11039, 1:500, RRID: AB_2534096), Alexa Fluor 594 goat anti-guinea pig (Invitrogen, A-11076, 1:500, RRID: AB_2534120), Alexa Fluor 647 goat anti-rabbit (Invitrogen, A-21245, 1:500, RRID: AB_2535813), Alexa Fluor 488 donkey anti-rabbit (Invitrogen, A-21206, 1:500, RRID: AB_2535792), Alexa Fluor 594 donkey anti-rat (Invitrogen, A-21209, 1:500, RRID: AB_2535795). Nuclei were counterstained with DAPI.

For immunofluorescence staining of rat brain tissue, 5 µm-thick paraffin sections were deparaffinized, rehydrated, and subjected to antigen retrieval by boiling in 10 mM sodium citrate buffer with 0.05%

Tween-20 (pH 6.0) for 10 min. The remaining staining procedure was performed as described above for the organoid sections.

Confocal microscopy and image analysis

To evaluate the density of excitatory synapses, images were acquired using a Leica SP8X confocal microscope with 60x magnification, 4x digital zoom, 0.3 μm z-step size, and Lightning mode for deconvolution. At least four random regions per organoid and three to four regions per rat brain sample were imaged. Pre- and post-synaptic puncta were identified using the "Spots" identifier in Imaris software (Bitplane, v9.7.2) with background subtraction enabled. The average diameter of randomly selected puncta was measured and applied for spot detection. Detection parameters were set to an xy diameter of 0.5 μm and z diameter of 1 μm for pre-synaptic puncta and an xy diameter of 0.4 μm and z diameter of 0.8 μm for post-synaptic puncta. Artifact spots not representing synaptic puncta were excluded by adjusting the "Quality" and "Intensity StdDev" thresholds. The "Colocalize Spots" function was used to identify adjacent pre- and post- synaptic puncta within a distance of 0.5 μm. Neuronal processes, delineated using the "Surfaces" function based on TUBB3 staining, were used to normalize synaptic density measurements.

Cell densities in VZ were quantified by acquiring images around the VZ using a Leica SP8X confocal microscope with 40x magnification. Image analysis was performed with ImageJ. Nuclei segmentation was performed using the StarDist ImageJ plugin on DAPI channel with default settings. A ROI encompassing the VZ was defined in each image based on cell orientation and the density of DAPI$^+$, PAX6$^+$ and CTIP2$^+$ cells. Only nuclei within the ROI were included for intensity measurements in PAX6 and CTIP2 channels. Cells were classified as PAX6$^+$ and CTIP2$^+$ if their mean fluorescence intensity above a specific threshold. Threshold values were adjusted to optimize the detection of visually identifiable PAX6$^+$ and CTIP2$^+$ cells while minimizing false positives.

Supplemental references

Li, C., Fleck, J. S., Martins-Costa, C., Burkard, T. R., Themann, J., Stuempflen, M., Peer, A. M., Vertesy, Á., Littleboy, J. B., Esk, C., Elling, U., Kasprian, G., Corsini, N. S., Treutlein, B., & Knoblich, J. A. (2023). Single-cell brain organoid screening identifies developmental defects in autism. *Nature*, *621*(7978), 373–380. https://doi.org/10.1038/s41586-023-06473-y

Voshart, D. C., Klaver, M., Jiang, Y., van Weering, H. R. J., van Buuren-Broek, F., van der Linden, G. P., Cinat, D., Kiewiet, H. H., Malimban, J., Vazquez-Matias, D. A., Reali Nazario, L., Scholma, A. C., Sewdihal, J., van Goethem, M.-J., van Luijk, P., Coppes, R. P., & Barazzuol, L. (2024). Proton therapy induces a local microglial neuroimmune response. *Radiotherapy and Oncology*, *193*(Accepted), 110117. https://doi.org/10.1016/j.radonc.2024.110117
