# Supplementary material for: Differential synaptic signaling responses in human cortical organoids after photon and proton irradiation
Source: Stem Cell Reports. 2026 Jan 8;21(2):102777. doi: 10.1016/j.stemcr.2025.102777 (PMC12903084; doi:10.1016/j.stemcr.2025.102777)
Supplement: Document S2. Article plus supplemental information [file mmc3.pdf]

STEM CELL REPORTS

Report

OPEN ACCESS

Differential synaptic signaling responses in human cortical organoids after photon and proton irradiation

Yuting Jiang,[1,2] Danieli Born Guerra,[1,2,4] Daniëlle C. Voshart,[1,2,4] Eline Hageman,[1,2] Luiza Reali Nazario,[1,2] Marc-Jan van Goethem,[1,3] Rob P. Coppes,[1,2] and Lara Barazzuol[1,2,5,*]

[1]Department of Radiation Oncology, University Medical Center Groningen, University of Groningen, Groningen, the Netherlands
[2]Department of Biomedical Sciences, University Medical Center Groningen, University of Groningen, Groningen, the Netherlands
[3]Department of Biomedical Sciences, PARTREC, University Medical Center Groningen, University of Groningen, Groningen, the Netherlands
[4]These authors contributed equally
[5]Lead contact
*Correspondence: l.barazzuol@umcg.nl
https://doi.org/10.1016/j.stemcr.2025.102777

SUMMARY

Brain tumors are the most common solid cancer in children, with radiotherapy being a primary treatment. Proton therapy, with its precise dose distribution, is increasingly being used in these patients to minimize damage to the developing brain. However, the biological effects of proton irradiation on the human brain remain unclear. To investigate this, human cortical organoids were exposed to conventional photons, plateau protons, and spread-out Bragg peak (SOBP) protons, followed by comparative transcriptomic profiling. While photons and protons induced similar transcriptional profiles characterized by apoptosis and downregulation of DNA replication, SOBP protons uniquely downregulated genes involved in brain development and synaptic signaling. Functional calcium imaging, cell deconvolution analysis, and immunostaining indicated that SOBP protons impaired neural network function, due to reduced synaptic density and loss in excitatory neuron progenitors. These findings underscore the distinct biological effects of SOBP protons and their potential impact on the developing brain.

INTRODUCTION

Brain and central nervous system (CNS) tumors are the most common type of solid tumors in children and young adolescents (Siegel et al., 2023). Despite the heterogeneity of tumor types, radiotherapy remains a primary treatment modality in the management of many pediatric brain tumors. Compared to conventional photon-based radiotherapy, proton therapy offers advantages in dose distribution due to its unique physical properties, allowing more healthy tissue to be spared (Mohan and Grosshans, 2017). Unlike photons, protons lose only a small amount of energy in the entrance region, referred to as the plateau protons, and release all their remaining energy at the end of their path in a narrow peak known as the Bragg peak (Baumann et al., 2016; Gondi et al., 2016; Mohan and Grosshans, 2017; Padovani et al., 2012). By combining multiple Bragg peaks of varying energies and intensities, a spread-out Bragg peak (SOBP) can be created to cover the entire tumor volume (Daugherty et al., 2013; DeNunzio and Yock, 2020). The energy deposition drops dramatically after the peak, thus reducing radiation exposure to surrounding healthy tissues (DeNunzio and Yock, 2020). As proton therapy becomes more accessible, it has emerged as a favored treatment option for pediatric brain tumors, offering improved dose conformation and reduced radiation-induced adverse effects (Baumann et al., 2020; DeNunzio and Yock, 2020; Gondi et al., 2016).

With advances in treatment, the 5-year survival rate for pediatric brain tumor patients has increased to approximately 75% (Siegel et al., 2023). However, these survivors usually experience long-term cognitive impairment and IQ decline due to radiation exposure (Al Dahhan et al., 2022; Gondi et al., 2016; Zhang et al., 2018), which significantly impact their academic achievements, career prospects, and independence (Kunin-Batson et al., 2011; Mabbott et al., 2005). Currently, no effective treatments exist to prevent these adverse effects (Simó et al., 2024; Turnquist et al., 2020), highlighting the need to understand the cellular and molecular changes in the developing brain that drive radiation-induced cognitive dysfunction. Besides, most studies addressing these questions have focused on photon irradiation, with limited research on plateau or SOBP protons. Although proton therapy treatment planning assumes a relative biological effectiveness (RBE) of 1.1, it remains unclear whether photons and protons induce comparable biological effects on the brain, especially in the Bragg peak, where the linear energy transfer (LET) is much higher compared to the plateau region (Mohan and Grosshans, 2017; Sørensen et al., 2021). While most healthy tissue is exposed to plateau protons during proton therapy, tissue adjacent to the tumor may also receive SOBP protons, raising questions about whether SOBP exposure affects normal tissue differently than photons or plateau protons.

© 2025 The Author(s).
Published by Elsevier Inc. on behalf of International Society for Stem Cell Research.
This is an open access article under the CC BY license (http://creativecommons.org/licenses/by/4.0/).

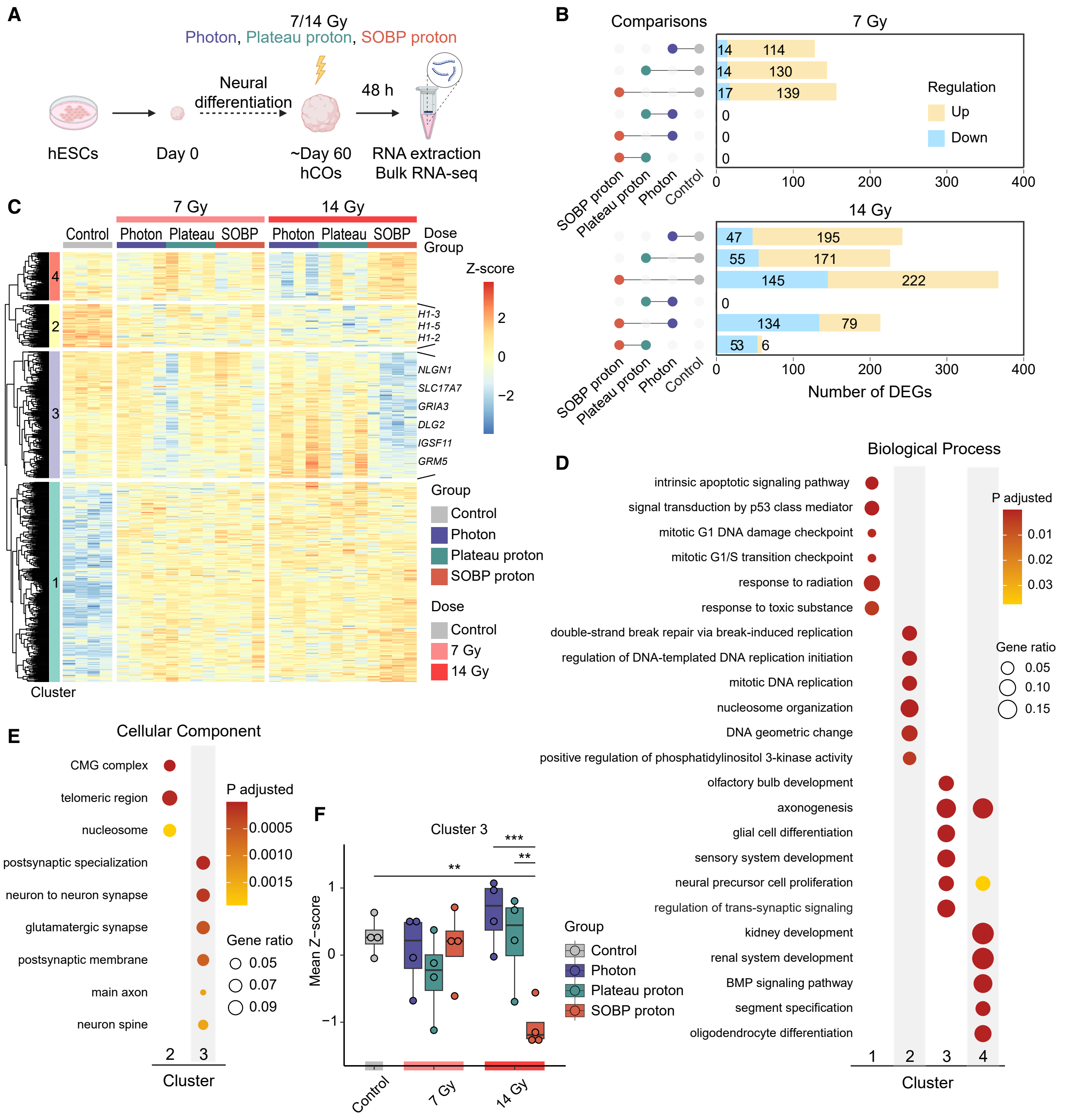

Figure 1. Comparative transcriptomic analysis of hCOs reveals unique effects of 14 Gy SOBP proton irradiation on brain development and synaptic signaling

(A) Schematic diagram of the experiment.

(B) Bar plot showing the number of DEGs across the different treatment groups among all the comparisons within the same radiation dose.

(C) Heatmap with Manhattan distance-based hierarchical clustering analysis of all DEGs detected from all the comparisons in (B). 4 main clusters are identified.

(D and E) Dot plots depicting the top six enriched GO terms for biological process (D) and cellular component (E) per cluster.

(legend continued on next page)

Animal models are widely used in preclinical research to study disease mechanisms; however, they have significant limitations in recapitulating the species-specific aspects of human brain development and tissue architecture (Eichmüller and Knoblich, 2022). While patient-derived materials, such as biopsies and postmortem brain tissues, can capture human-specific changes, they only provide limited snapshots of disease progression and are very difficult to obtain (Eichmüller and Knoblich, 2022). Human brain organoids, differentiated from human pluripotent stem cells (hPSCs), offer a promising model alternative. By applying exogenous cues that mimic endogenous patterning, brain region-specific organoids can be generated with cellular compositions and electrophysiological activities similar to those in the developing human brain (Trujillo et al., 2019; Velasco et al., 2019). These features have made brain organoids an increasingly valuable tool for modeling a range of neurodevelopmental conditions (Birey et al., 2022; Li et al., 2023; Di Lullo and Kriegstein, 2017; Samarasinghe et al., 2021; Sebastian et al., 2023).

In this study, we employ a human cortical organoid model to examine the effect of irradiation with photons, plateau protons, and SOBP protons on developing brain tissue. Our findings indicate that while photons and protons induce similar transcriptomic profiles characterized by increased apoptosis and cell-cycle arrest, SOBP protons uniquely downregulate genes involved in brain development and synaptic signaling. Further validation with live-cell calcium imaging recording, cell deconvolution analysis, and immunostaining suggests that reduced synaptic density, along with a decreased number of excitatory neural progenitors, likely contribute to the impaired neural network function observed after SOBP proton irradiation.

RESULTS

RNA sequencing of human cortical organoids reveals similar transcriptional responses to photon and plateau proton irradiation

To investigate how different types of radiation affect the developing brain, we generated human cortical organoids (hCOs) from human embryonic stem cells (hESCs) following an established protocol (Sloan et al., 2018). At around 60 days of differentiation, the organoids were irradiated with photons, plateau protons, or SOBP protons at doses of 7 and 14 Gy. These doses correspond to fractionated schedules of 15.75 and 56 Gy, respectively, delivered in 2 Gy fractions, with the latter representing a clinically relevant dose (Fowler, 1989). We then assessed transcriptional changes 48 h post-irradiation using bulk RNA sequencing (RNA-seq) (Figure 1A).

To identify the biological processes affected by the different radiation types, we performed differential gene expression analysis across the different treatment groups. A dose-dependent increase in the number of differentially expressed genes (DEGs) was observed across the comparisons (Figure 1B). In line with our previous findings in adult rat brains (Voshart et al., 2024), photon and plateau proton irradiation induced similar transcriptional changes in hCOs, with no DEGs observed between these groups at either dose level (Figure 1B). While SOBP proton irradiation exhibited a transcriptional profile similar to the other groups irradiated with 7 Gy, it uniquely downregulated more genes at a higher dose of 14 Gy compared to photon or plateau proton irradiation (Figure 1B).

DEGs from all the comparisons were then divided into four clusters using Manhattan distance-based hierarchical clustering (Figure 1C) and annotated per cluster with Gene Ontology (GO) analysis (Figures 1D, 1E, and S1A). Clusters 1 and 2 comprised genes that were either upregulated or downregulated after irradiation in a dose-dependent manner, respectively, regardless of the radiation type (Figures 1C and S1B). Enriched biological processes indicated that the upregulated genes were associated with terms related to p53-mediated apoptotic pathways, while the downregulated genes were associated with terms related to DNA replication and nucleosome organization (Figure 1D). Cluster 4 included a small set of genes induced by 14 Gy SOBP proton irradiation while being downregulated by 14 Gy photon irradiation (Figures 1C and S1B), with GO terms related to development and BMP signaling pathway (Figure 1D).

SOBP proton irradiation leads to a deregulation of brain development and synaptic signaling

In contrast, cluster 3 contained genes uniquely downregulated by 14 Gy SOBP proton irradiation but not by other radiation types (Figure 1F). These genes were associated with brain development processes, such as "olfactory bulb development," "axonogenesis," and "glial cell differentiation" (Figure 1D). Notably, about one-fourth of the genes in cluster 3 were synaptic genes as identified by SYNGO enrichment analysis (Table S1) (Koopmans et al., 2019), including *SLC17A7*, which encodes the pre-synaptic

(F) Boxplots showing the mean *Z* score of genes in cluster 3 for each condition. Boxes are drawn from first quartile to third quartile, with horizontal lines indicating the median. $n = 4$ pools per group, each pool contains 3–4 organoids. $^{**}p < 0.01$ and $^{***}p < 0.001$. Two-way ANOVA followed by Tukey's multiple comparisons test was used for data shown in (F).
See also Figure S1 and Table S1.

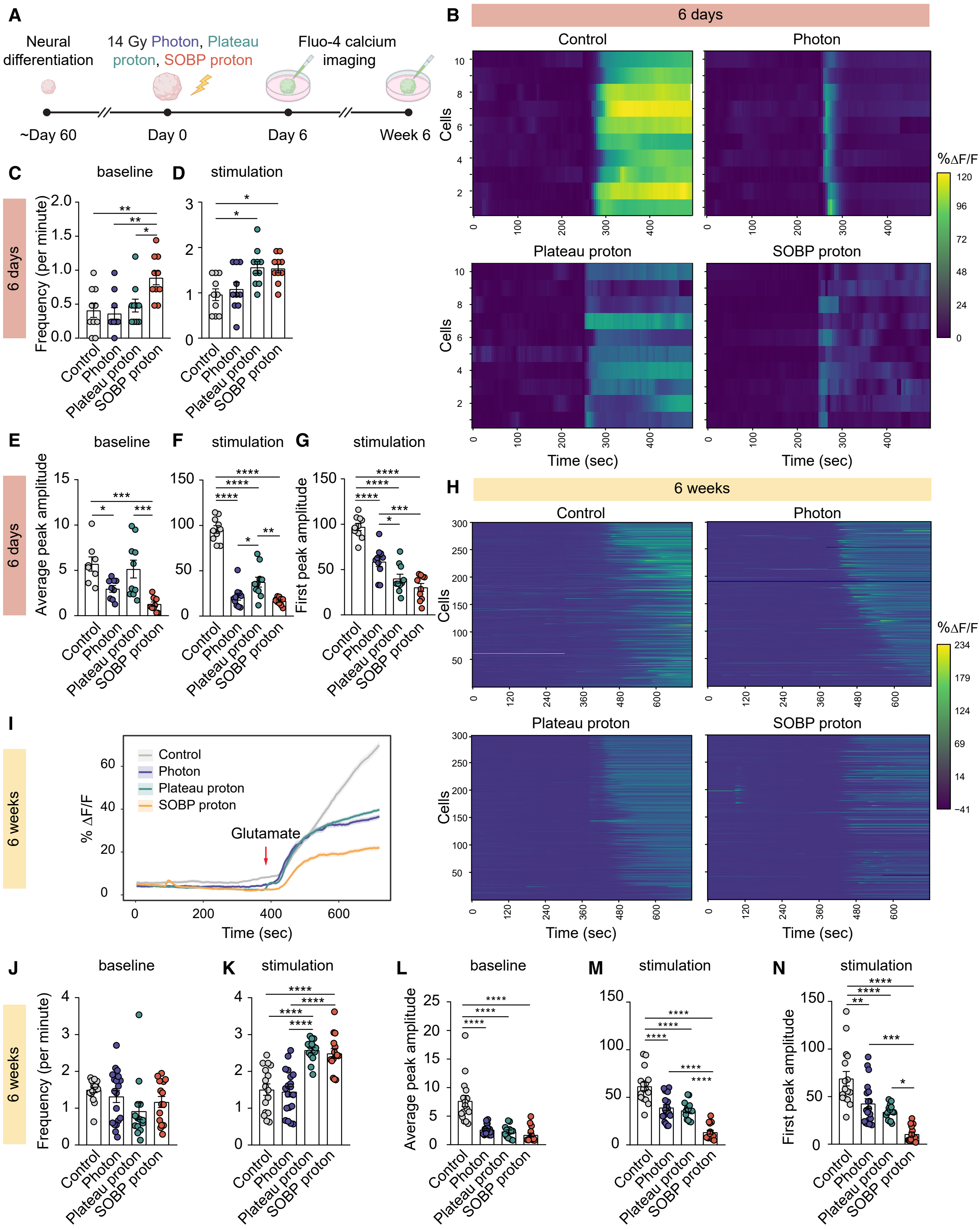

(legend on next page)

glutamate transporter VGLUT1, as well as *GRIA3* and *GRM5*, which encode different glutamate receptors (Figure 1C). GO analysis further confirmed disrupted synaptic function following 14 Gy SOBP proton irradiation, with enrichment in biological processes related to "regulation of *trans*-synaptic signaling" and cellular components such as "postsynaptic specialization" and "neuron to neuron synapse" (Figures 1D and 1E).

Overall, these results suggest that while photon and plateau proton irradiation induce similar transcriptional changes in hCOs characterized by a shared upregulation of apoptosis and downregulation of DNA replication, 14 Gy SOBP proton irradiation has a more distinct impact on brain development and synaptic signaling.

SOBP proton irradiation more severely impairs neural network function

To validate whether the observed transcriptional changes led to functional alterations in neuronal network over time, we performed live-cell calcium imaging at 6 days and 6 weeks after 14 Gy photon, plateau proton, and SOBP proton irradiation (Figure 2A). hCOs were loaded with the calcium-sensitive dye Fluo-4, and calcium dynamics were recorded before and after the addition of glutamate, the major excitatory neurotransmitter in the brain. Changes in fluorescence intensity over time ($\Delta F/F(t)$) for individual cells were plotted to assess neural activity (Figures 2B, 2H, and S2). At 6 days post-irradiation, photon exposure slightly reduced the amplitude of spontaneous calcium transients without altering their frequency, whereas plateau protons did not affect either frequency or amplitude (Figures 2C and 2E). SOBP proton irradiation, in contrast, resulted in more frequent but smaller transients compared to control and plateau proton-irradiated groups (Figures 2C and 2E). Upon glutamate stimulation, both plateau and SOBP proton irradiation increased the frequency of calcium transients (Figure 2D), while all radiation types reduced their peak amplitude (Figures 2F and 2G). At 6 weeks post-irradiation, the effect of SOBP proton irradiation on neuronal activity became more pronounced, as reflected in the averaged calcium transients (Figure 2I). Although no difference was observed in the frequency of spontaneous calcium transients across groups, all radiation types reduced their amplitude (Figures 2J and 2L). Interestingly, the increased frequency of calcium transients upon glutamate stimulation seen after plateau and SOBP proton irradiation at 6 days (Figure 2D) became more evident at 6 weeks (Figure 2K). Consistent with the early time point, all radiation types reduced the peak amplitude of glutamate-induced calcium transients at 6 weeks, with SOBP proton irradiation exerting the most pronounced effect (Figures 2M and 2N). This reduction in neuronal activity aligns with the transcriptional changes observed in synaptic genes, particularly those involved in glutamatergic signaling following SOBP proton irradiation (Figure 1E). In conclusion, these findings indicate that 14 Gy SOBP proton irradiation has a more profound effect on neural network activity compared to control group and irradiation with other radiation qualities.

Reduced synaptic density may underlie impaired neural network function following SOBP proton irradiation

Given the transcriptional and functional evidence of disrupted synaptic signaling following 14 Gy SOBP proton irradiation, we further validated these findings using immunostaining for VGLUT1, a presynaptic vesicular glutamate transporter, and HOMER1, a postsynaptic density

Figure 2. More pronounced impairment of neural activity after SOBP proton irradiation

(A) Schematic diagram of the experiment.

(B) Heatmap showing spontaneous and glutamate-induced calcium transients in 10 individual cells per group at 6 days post-irradiation.

(C and D) Quantification of spontaneous (C) and glutamate-induced (D) calcium transient frequency (events per minute) in organoids at 6 days post-irradiation.

(E and F) Quantification of average calcium transient peak amplitudes during baseline (E) and after glutamate stimulation (F) in organoids at 6 days post-irradiation.

(G) Quantification of the first calcium transient peak amplitude following glutamate stimulation in organoids at 6 days post-irradiation.

(H) Heatmap showing spontaneous and glutamate-induced calcium transients in 300 individual cells per group at 6 weeks post-irradiation.

(I) Averaged calcium traces from 300 cells per group at 6 weeks post-irradiation before and after glutamate stimulation. Lines represent the mean, and shaded areas indicate the SEM. The red arrow indicates the addition of glutamate.

(J and K) Quantification of spontaneous (J) and glutamate-induced (K) calcium transient frequency (events per minute) in organoids at 6 weeks post-irradiation.

(L and M) Quantification of average calcium transient peak amplitudes during baseline (L) and after glutamate stimulation (M) in organoids at 6 weeks post-irradiation.

(N) Quantification of the first calcium transient peak amplitude following glutamate stimulation in organoids at 6 weeks post-irradiation. n = 10 cells per group (C–G); n = 14–19 cells per group (J–N). Scatterplots show mean ± SEM (C–G and J–N). $^{*}p < 0.05$, $^{**}p < 0.01$, $^{***}p < 0.001$, and $^{****}p < 0.0001$. One-way ANOVA followed by Tukey's multiple comparisons test was used for data shown in (C)–(G) and (J)–(N). See also Figure S2.

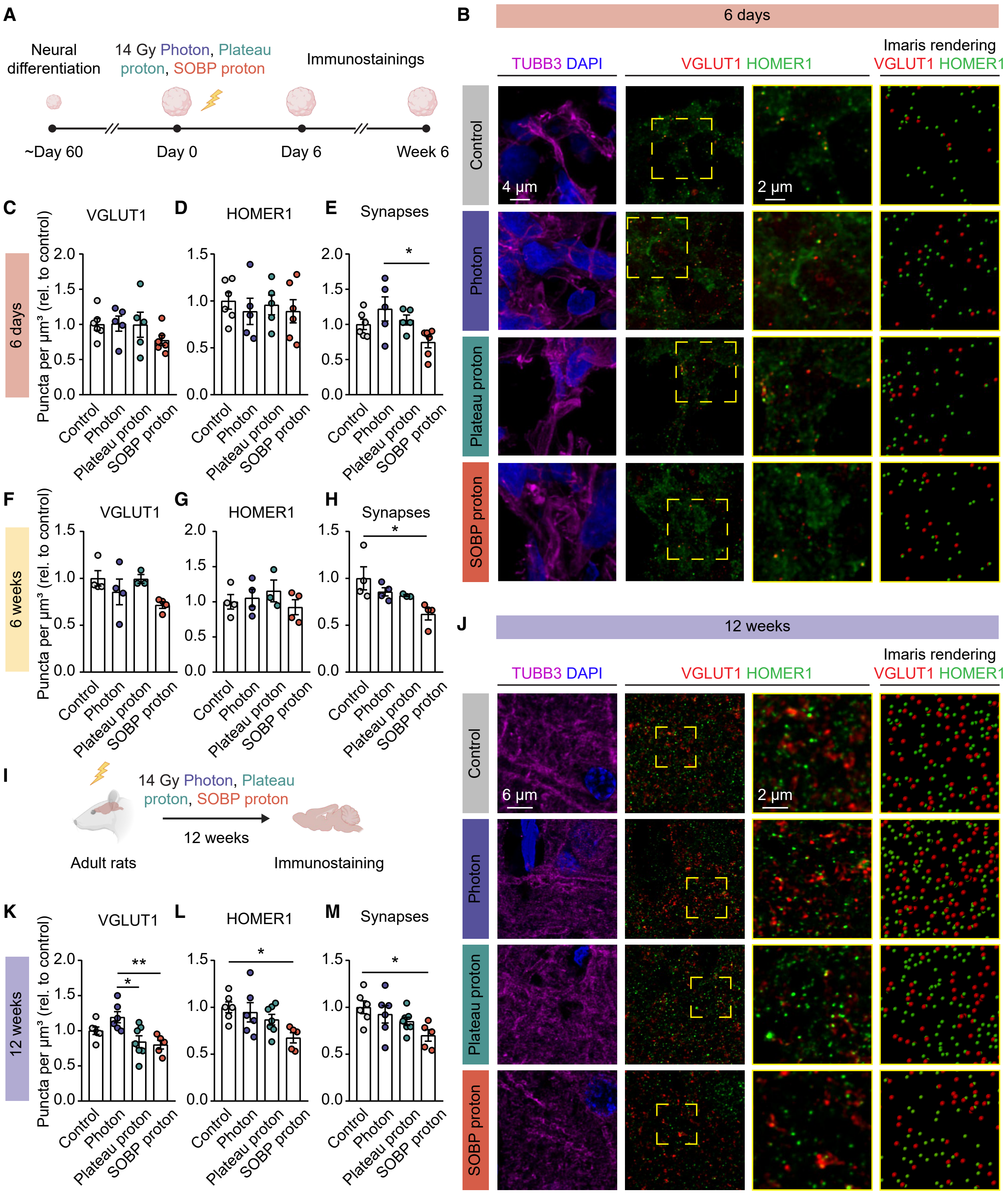

(legend on next page)

scaffold protein, at both 6 days and 6 weeks post-irradiation (Figures 3A–3H). These proteins, both components of glutamatergic synapses, were quantified to assess synaptic density. No significant differences in VGLUT1 and HOMER1 density were observed between groups at either time point (Figures 3B–3D and 3F–3G). Although no reduction in synaptic density was detected at 6 days post-irradiation compared with the control (Figure 3E), SOBP proton irradiation significantly reduced synaptic density at 6 weeks, as indicated by decreased co-localization of VGLUT1 and HOMER1 puncta relative to the control group (Figure 3H). To determine whether SOBP proton irradiation induces a similar effect *in vivo*, we performed the same staining in adult rat brains 12 weeks after 14 Gy photon, plateau, or SOBP proton irradiation (Figures 3I and 3J). While radiation did not affect VGLUT1 density compared with control, SOBP protons significantly reduced HOMER1 expression and synaptic density in the cortex (Figures 3K–3M). Overall, these findings suggest that the reduction in synaptic number may contribute to the more pronounced neural network impairment seen after SOBP proton irradiation.

SOBP proton irradiation reduces dorsal forebrain progenitor cells while increasing astrocyte proportion

In the developing cerebral cortex, excitatory neurons arise from dorsal forebrain progenitors, while interneurons are mostly generated by progenitors in the ventral forebrain and later migrate to the cortical plate (Eichmüller and Knoblich, 2022). Since transcriptionally SOBP proton irradiation affects many brain development-related genes (Figure 1D), we examined whether different types of radiation also differentially affect regional brain identities. Irradiation with 14 Gy SOBP protons seems to decrease the expression of many dorsal forebrain markers at 48 h post-irradiation (Figure 4A). To gain further insight into how radiation affects individual cell types, we deconvolved our bulk RNA-seq data using CIBERSORTx (Newman et al., 2019). Photon irradiation significantly increases the proportion of interneuron precursor cells (INPs) compared to unexposed controls (Figures 4B and 4C). Additionally, 14 Gy SOBP irradiation appears to result in an increase in astrocytes (Figures 4B and 4C). We next investigated the impact of 14 Gy radiation at 6 days post-irradiation on the cellular composition of the ventricular zone (VZ), a region important for neurogenesis in the dorsal forebrain, with immunostaining for the dorsal forebrain neural progenitor cell marker PAX6 and deep-layer neuron marker CTIP2 (Figures 4D–4G). We found a significant decrease in $PAX6^{+}$ cells within the VZ after SOBP proton irradiation (Figures 4D and 4E), with no significant differences in the percentage of $CTIP2^{+}$ cells or total cell density (Figures 4F and 4G). Our findings suggest that SOBP proton irradiation leads to an increase in astrocytes and a decrease in dorsal forebrain progenitors within the VZ. These changes in cell composition may ultimately disrupt neural network function and brain development.

DISCUSSION

Clinical evidence, though mainly derived from small and non-randomized studies, suggests that proton therapy may improve cognitive outcomes compared to photon therapy, primarily owing to its more precise physical dose distribution (Mash et al., 2023; Sienna et al., 2024). Despite its widespread use as a preferred treatment modality for pediatric brain and CNS tumors, uncertainties persist regarding the biological and immunological differences between proton and photon radiation, as a number of studies have reported

Figure 3. Changes in synaptic density after photon, plateau proton, and SOBP proton irradiation
(A) Schematic diagram of the experiment.
(B) Representative images of pre-synaptic protein VGLUT1 and post-synaptic protein HOMER1 in $TUBB3^{+}$ neurons from 6-day post-irradiation organoids. The yellow dashed boxes are magnified in the images next to them. The final images show the 3D rendered VLGUT1 and HOMER1 puncta from insets using Imaris software.
(C–E) Quantification of VGLUT1 puncta density (C), HOMER1 puncta density (D), and synaptic density (E) in 6-day post-irradiation organoids.
(F–H) Quantification of VGLUT1 puncta density (F), HOMER1 puncta density (G), and synaptic density (H) in 6-week post-irradiation organoids.
(I) Adult rats received 14 Gy photon, plateau proton, or SOBP proton irradiation and were sacrificed 12 weeks later.
(J) Representative images of pre-synaptic protein VGLUT1 and post-synaptic protein HOMER1 in $TUBB3^{+}$ neurons from the cortex of rat brains 12 weeks post-irradiation. The yellow dashed boxes are magnified in the images next to them. The final images show the 3D rendered VLGUT1 and HOMER1 puncta from insets using Imaris software.
(K–M) Quantification of VGLUT1 puncta density (K), HOMER1 puncta density (L), and synaptic density (M) in the cortex of rat brains 12 weeks post-irradiation.
n = 5–6 organoids per group (C–E). n = 3–4 organoids per group (F–H). n = 5–7 animals per group (K–M). Bar plots show mean ± SEM (C–E, F–H, and K–M). $^{*}p < 0.05$, $^{**}p < 0.01$. One-way ANOVA followed by Tukey's multiple comparisons test was used for data shown in (C)–(E), (F)–(H), and (K)–(M).

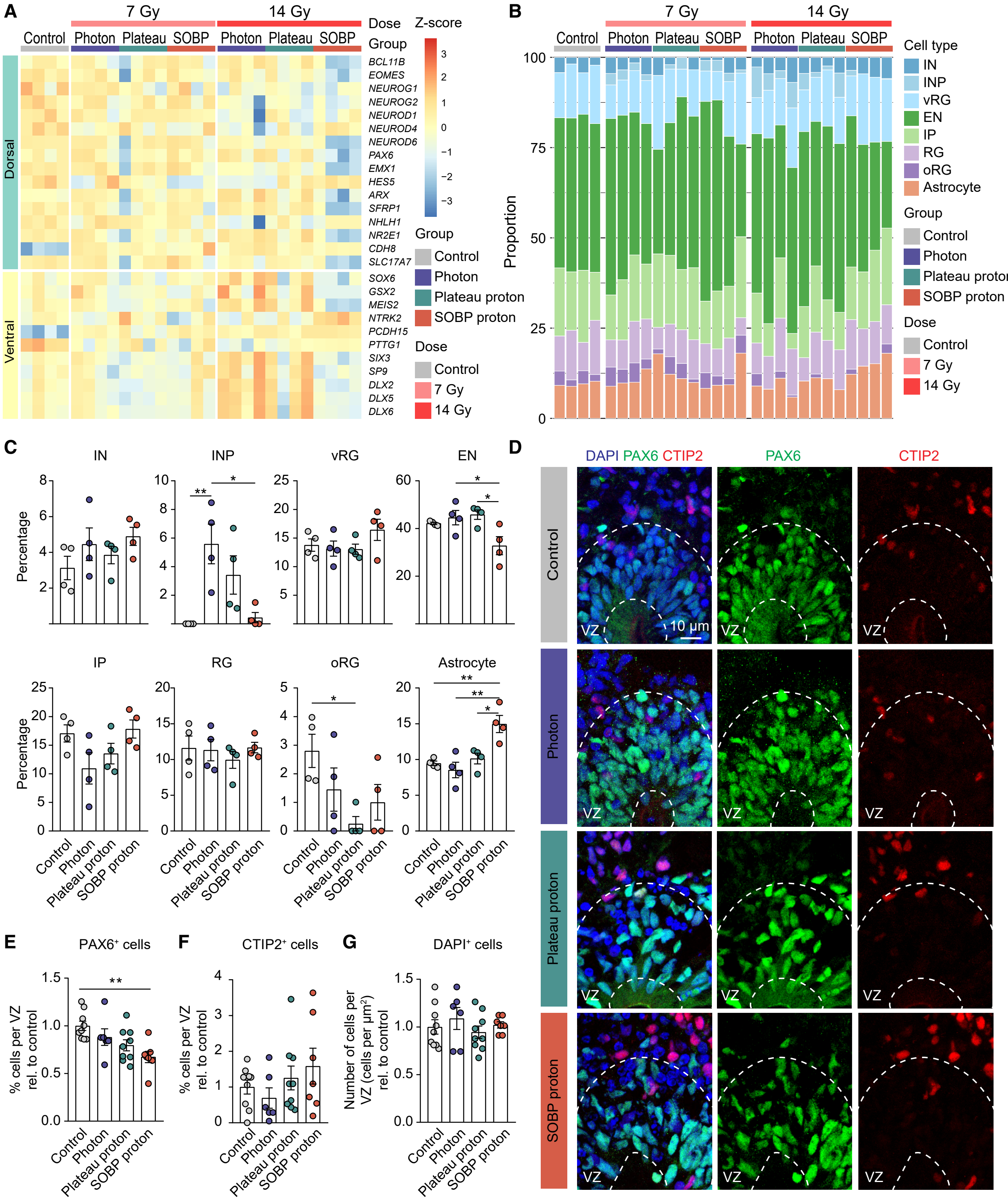

Figure 4. SOBP protons seem to decrease the proportion of neuronal progenitors while increasing astrocytes

(A) Heatmap depicting expressions of differentially expressed regional brain identity markers.

(B) Barplot showing the proportions of cell types in hCOs following cell deconvolution.

(legend continued on next page)

an increased incidence of imaging changes in patients after proton therapy (Gunther et al., 2015; Harrabi et al., 2022; Ludmir et al., 2019). Preclinical studies investigating the effects of photon and proton irradiation on normal brain tissue at the transcriptomic level are limited, with even fewer studies directly comparing plateau protons and SOBP protons. This gap of knowledge is particularly relevant, as normal tissue adjacent to the tumor may be exposed to SOBP protons, which are assumed to have higher RBE due to increased LET and, consequently, greater normal tissue damage than plateau protons received by most co-irradiated normal tissues.

A recent study demonstrated that 3 Gy photons and SOBP protons induce comparable early apoptotic responses in the neonatal mouse hippocampus and cerebellum (Giovannini et al., 2024). Consistent with this, our transcriptional data from the *in vitro* hCO model revealed that both radiation types induce apoptosis and cell-cycle arrest at similar levels in a dose-dependent manner. Moreover, our findings align with previous research showing that photon and plateau proton irradiation induce similar transcriptional profiles in the adult rat brain months after radiation exposure (Voshart et al., 2024). Similarly, our hCO model demonstrated early shared transcriptional changes between these two radiation types, suggesting a potential similarity in their effects and underscoring the utility of the hCO model for studying radiation effects on the developing brain.

Clinically, a fixed RBE of 1.1 is applied for proton therapy to account for its higher cell killing efficiency compared to photons (Paganetti et al., 2002; Sørensen et al., 2021). However, in practice, RBE is not constant and varies depending on factors such as tissue type, LET, fractionation dose, and biological endpoints (Sørensen et al., 2021; Zhou et al., 2024). Notably, an increase in RBE has been observed at the distal edge of the SOBP, a region where healthy tissues or critical organs are often located. This variability raises concerns, as clinical studies have reported radiation-induced imaging changes in this area (Bertolet et al., 2022; Engeseth et al., 2021; Winter et al., 2024). Cognitive dysfunction is a common long-term side effect of radiotherapy. Studies suggest that persistent radiation-induced changes in synaptic proteins, dendritic morphology, and spine density could contribute to cognitive decline (Parihar and Limoli, 2013; Parihar et al., 2015; Wu et al., 2022). While our study did not specifically investigate the distal edge of the SOBP, the pronounced effect of 14 Gy SOBP protons on brain development and synaptic signaling highlights the importance of refining proton therapy parameters, such as dose and energy distribution, to mitigate potential adverse effects such as (symptomatic) imaging changes and cognitive impairment in the developing brain.

Synapse formation during early brain development is essential for learning and memory. Disruptions in synapses or synapse-related genes and proteins have been associated with many neurodevelopmental, neurodegenerative, and psychiatric diseases (Dejanovic et al., 2024; Michetti et al., 2022; Wang et al., 2018). Alongside, recent studies have shown that brain tumor cells can hijack synaptic plasticity mechanisms leading to neuronal hyperexcitability, thus enhancing tumor growth and survival (Taylor et al., 2023). Our findings demonstrate that SOBP protons exert a more substantial impact on neuronal network function, raising the possibility that proton therapy may be more effective at disrupting neuron-to-glioma synapses, thus enhancing tumor control. Future investigations integrating hCO models with brain tumor organoids could help address this important question.

Our previous work also found that SOBP protons induce a greater expression of inflammation-related microglial priming genes than photons and plateau protons. However, the current hCO model lacks microglia, limiting our ability to investigate the interplay between microglia and neurons under different radiation conditions. Emerging techniques for generating brain organoids with integrated microglia could provide a more comprehensive understanding of radiation-induced biological changes in the developing brain (Sabogal-Guaqueta et al., 2024; Zhang et al., 2023).

Together, our data provide novel insights into the early responses of the human developing cortex to different types of radiation using hCOs as a model. While photon and plateau proton radiation lead to similar transcriptional profiles, SOBP proton radiation has a more pronounced impact on neural network function. Further investigations are needed to determine whether these effects translate into long-term functional consequences in the developing brain *in vivo*.

MATERIALS AND METHODS

Due to word limitation, a detailed description of the methods is provided in the supplemental methods.

(C) Individual bar plots illustrating changes in the percentage of each cell type following 14 Gy irradiation as shown in (B).
(D) Representative images of PAX6 and CTIP2 with annotated ventricular zone (VZ).
(E–G) Quantification of the percentage of $PAX6^+$ cells (E), $CTIP2^+$ cells (F), and the density of $DAPI^+$ cells in VZ (G). $n = 6–9$ VZ per group. Bar plots show mean ± SEM (C, E, F, and G). $^*p < 0.05$, $^{**}p < 0.01$. One-way ANOVA followed by Tukey's multiple comparisons test was used for data shown in (C), (E), (F), and (G). IN, interneuron; INP, interneuron precursor cell; vRG, ventral radial glial cell; EN, excitatory neuron; IP, intermediate progenitor cell; RG, radial glial cell; oRG, outer radial glial cell.

hCO generation and irradiation

hCOs were generated following an established protocol (Sloan et al., 2018). Details are provided in the supplemental methods.

After approximately 60 days of differentiation, hCOs were irradiated with either 7 or 14 Gy of radiation. Photon irradiation was performed with Cesium-137 source (662 keV, dose rate 0.59 Gy/min) at the Department of Biomedical Sciences of UCMG. Proton irradiation, including both plateau protons (scattered beam, 150 MeV shoot-through, LET 0.5 keV/μm, dose rate 4 Gy/min) and SOBP protons (150 MeV, LET 3.1 keV/μm, dose rate 6 Gy/min), was conducted at the Particle Therapy Research Center (PARTREC) accelerator facility of the UMCG.

Animal irradiation

Adult male Wistar (Hsd/Cpb:WU) rats were irradiated with 14 Gy photons or protons and sacrificed 12 weeks later. Details are provided in the supplemental methods.

Bulk RNA-seq

RNA was isolated from hCOs 48 h after irradiation. For RNA isolation, library preparation, sequencing, and data analysis, see supplemental methods.

Calcium imaging

Calcium activity in hCOs was visualized 6 days and 6 weeks after irradiation using the Fluo-4 Direct Calcium Kit (see supplemental methods).

Immunofluorescence staining

hCOs were collected 6 days and 6 weeks post-irradiation for immunofluorescence staining. Details of the staining procedure, confocal imaging, and image analysis are provided in the supplemental methods.

Statistical analysis

Statistical analysis was conducted using GraphPad Prism 8. Two-way analysis of variance (ANOVA) with Tukey's multiple comparison test was applied to analyze statistical differences in mean Z score from RNA-seq data across groups within each radiation dose. For calcium imaging, immunofluorescence quantification, and cell proportion analysis from cell deconvolution data, one-way ANOVA with Tukey's multiple comparison test was used. Statistical significance was defined as $p < 0.05$.

RESOURCE AVAILABILITY

Lead contact

Further information and requests for resources reported in this paper should be directed to the lead contact, Lara Barazzuol (l.barazzuol@umcg.nl).

Materials availability

This study did not generate any new reagents.

Data and code availability

The accession number for the RNA-seq data reported in this paper is GEO: GSE311001. This paper does not generate any original code. Script and code can be requested from the lead contact.

ACKNOWLEDGMENTS

This work was supported by KWF Kankerbestrijding (project no. 11148 to L.B.), Stand Up To Cancer (SU2C) and Cancer Research UK (CRUK) Pediatric Cancer New Discoveries Challenge Team Grant (project no. SU2C#RT6186 to L.B.), Stichting De Cock-Hadders (project no. 2022-28 to D.C.V.), and China Scholarship Council (project no. 201906320080 to Y.J.). Some figures were created using BioRender.com.

AUTHOR CONTRIBUTIONS

Conceptualization, L.B.; methodology, Y.J., D.B.G., D.C.V., and E.H.; investigation, Y.J., D.B.G., and E.H.; resources, D.C.V. and L.R.N.; writing – original draft, Y.J.; writing – review and editing, D.B.G., D.C.V., E.H., L.B., and R.P.C.; visualization, Y.J. and D.B.G.; supervision, L.B. and R.P.C.; funding acquisition, L.B. and D.C.V.

DECLARATION OF INTERESTS

The authors declare no competing interests.

DECLARATION OF GENERATIVE AI AND AI-ASSISTED TECHNOLOGIES IN THE WRITING PROCESS

During the preparation of this work, the first author used ChatGPT in order to improve readability. After using this tool, the authors reviewed and edited the content as needed and take full responsibility for the content of the publication.

SUPPLEMENTAL INFORMATION

Supplemental information can be found online at https://doi.org/10.1016/j.stemcr.2025.102777.

Received: January 15, 2025
Revised: December 4, 2025
Accepted: December 5, 2025
Published: January 8, 2026

REFERENCES

Baumann, B.C., Mitra, N., Harton, J.G., Xiao, Y., Wojcieszynski, A.P., Gabriel, P.E., Zhong, H., Geng, H., Doucette, A., Wei, J., et al. (2020). Comparative Effectiveness of Proton vs Photon Therapy as Part of Concurrent Chemoradiotherapy for Locally Advanced Cancer. JAMA Oncol. *6*, 237–246. https://doi.org/10.1001/jamaoncol.2019.4889.

Baumann, M., Krause, M., Overgaard, J., Debus, J., Bentzen, S.M., Daartz, J., Richter, C., Zips, D., and Bortfeld, T. (2016). Radiation

oncology in the era of precision medicine. Nat. Rev. Cancer *16*, 234–249. https://doi.org/10.1038/nrc.2016.18.

Bertolet, A., Abolfath, R., Carlson, D.J., Lustig, R.A., Hill-Kayser, C., Alonso-Basanta, M., and Carabe, A. (2022). Correlation of LET With MRI Changes in Brain and Potential Implications for Normal Tissue Complication Probability for Patients With Meningioma Treated With Pencil Beam Scanning Proton Therapy. Int. J. Radiat. Oncol. Biol. Phys. *112*, 237–246. https://doi.org/10.1016/j.ijrobp.2021.08.027.

Birey, F., Li, M.-Y., Gordon, A., Thete, M.V., Valencia, A.M., Revah, O., Paşca, A.M., Geschwind, D.H., and Paşca, S.P. (2022). Dissecting the molecular basis of human interneuron migration in forebrain assembloids from Timothy syndrome. Cell Stem Cell *29*, 248–264.e7. https://doi.org/10.1016/j.stem.2021.11.011.

Al Dahhan, N.Z., Cox, E., Nieman, B.J., and Mabbott, D.J. (2022). Cross-translational models of late-onset cognitive sequelae and their treatment in pediatric brain tumor survivors. Neuron *110*, 2215–2241. https://doi.org/10.1016/j.neuron.2022.04.009.

Daugherty, L.C., Fisher, B.J., Knowlton, C.A., Mackay, M.K., Wazer, D.E., Dragun, A.E., Brashears, J.H., Brashears, J.H., Brashears, J.H., Brashears, J.H., et al. (2013). Proton Therapy. In Encyclopedia of Radiation Oncology (Springer Berlin Heidelberg), pp. 675–690.

Dejanovic, B., Sheng, M., and Hanson, J.E. (2024). Targeting synapse function and loss for treatment of neurodegenerative diseases. Nat. Rev. Drug Discov. *23*, 23–42. https://doi.org/10.1038/s41573-023-00823-1.

DeNunzio, N.J., and Yock, T.I. (2020). Modern Radiotherapy for Pediatric Brain Tumors. Cancers *12*, 1533. https://doi.org/10.3390/cancers12061533.

Eichmüller, O.L., and Knoblich, J.A. (2022). Human cerebral organoids — a new tool for clinical neurology research. Nat. Rev. Neurol. *18*, 661–680. https://doi.org/10.1038/s41582-022-00723-9.

Engeseth, G.M., He, R., Mirkovic, D., Yepes, P., Mohamed, A.S.R., Stieb, S., Fuller, C.D., Wu, R., Zhang, X., Hysing, L.B., et al. (2021). Mixed Effect Modeling of Dose and Linear Energy Transfer Correlations With Brain Image Changes After Intensity Modulated Proton Therapy for Skull Base Head and Neck Cancer. Int. J. Radiat. Oncol. Biol. Phys. *111*, 684–692. https://doi.org/10.1016/j.ijrobp.2021.06.016.

Fowler, J.F. (1989). The linear-quadratic formula and progress in fractionated radiotherapy. Br. J. Radiol. *62*, 679–694. https://doi.org/10.1259/0007-1285-62-740-679.

Giovannini, D., Antonelli, F., Casciati, A., De Angelis, C., Denise Astorino, M., Bazzano, G., Fratini, E., Ampollini, A., Vadrucci, M., Cisbani, E., et al. (2024). Comparing the effects of irradiation with protons or photons on neonatal mouse brain: Apoptosis, oncogenesis and hippocampal alterations. Radiother. Oncol. *195*, 110267. https://doi.org/10.1016/j.radonc.2024.110267.

Gondi, V., Yock, T.I., and Mehta, M.P. (2016). Proton therapy for paediatric CNS tumours — improving treatment-related outcomes. Nat. Rev. Neurol. *12*, 334–345. https://doi.org/10.1038/nrneurol.2016.70.

Gunther, J.R., Sato, M., Chintagumpala, M., Ketonen, L., Jones, J.Y., Allen, P.K., Paulino, A.C., Okcu, M.F., Su, J.M., Weinberg, J., et al. (2015). Imaging Changes in Pediatric Intracranial Ependymoma Patients Treated With Proton Beam Radiation Therapy Compared to Intensity Modulated Radiation Therapy. Int. J. Radiat. Oncol. Biol. Phys. *93*, 54–63. https://doi.org/10.1016/j.ijrobp.2015.05.018.

Harrabi, S.B., von Nettelbladt, B., Gudden, C., Adeberg, S., Seidensaal, K., Bauer, J., Bahn, E., Mairani, A., Alber, M., Haberer, T., et al. (2022). Radiation induced contrast enhancement after proton beam therapy in patients with low grade glioma – How safe are protons? Radiother. Oncol. *167*, 211–218. https://doi.org/10.1016/j.radonc.2021.12.035.

Koopmans, F., van Nierop, P., Andres-Alonso, M., Byrnes, A., Cijsouw, T., Coba, M.P., Cornelisse, L.N., Farrell, R.J., Goldschmidt, H.L., Howrigan, D.P., et al. (2019). SynGO: An Evidence-Based, Expert-Curated Knowledge Base for the Synapse. Neuron *103*, 217–234.e4. https://doi.org/10.1016/j.neuron.2019.05.002.

Kunin-Batson, A., Kadan-Lottick, N., Zhu, L., Cox, C., Bordes-Edgar, V., Srivastava, D.K., Zeltzer, L., Robison, L.L., and Krull, K.R. (2011). Predictors of independent living status in adult survivors of childhood cancer: A report from the Childhood Cancer Survivor Study. Pediatr. Blood Cancer *57*, 1197–1203. https://doi.org/10.1002/pbc.22982.

Li, C., Fleck, J.S., Martins-Costa, C., Burkard, T.R., Themann, J., Stuempflen, M., Peer, A.M., Vertesy, Á., Littleboy, J.B., Esk, C., et al. (2023). Single-cell brain organoid screening identifies developmental defects in autism. Nature *621*, 373–380. https://doi.org/10.1038/s41586-023-06473-y.

Ludmir, E.B., Mahajan, A., Paulino, A.C., Jones, J.Y., Ketonen, L.M., Su, J.M., Grosshans, D.R., McAleer, M.F., McGovern, S.L., Lassen-Ramshad, Y.A., et al. (2019). Increased risk of pseudoprogression among pediatric low-grade glioma patients treated with proton versus photon radiotherapy. Neuro Oncol. *21*, 686–695. https://doi.org/10.1093/neuonc/noz042.

Di Lullo, E., and Kriegstein, A.R. (2017). The use of brain organoids to investigate neural development and disease. Nat. Rev. Neurosci. *18*, 573–584. https://doi.org/10.1038/nrn.2017.107.

Mabbott, D.J., Spiegler, B.J., Greenberg, M.L., Rutka, J.T., Hyder, D.J., and Bouffet, E. (2005). Serial Evaluation of Academic and Behavioral Outcome After Treatment With Cranial Radiation in Childhood. J. Clin. Oncol. *23*, 2256–2263. https://doi.org/10.1200/JCO.2005.01.158.

Mash, L.E., Kahalley, L.S., Raghubar, K.P., Goodrich-Hunsaker, N.J., Abildskov, T.J., De Leon, L.A., MacLeod, M., Stancel, H., Parsons, K., Biekman, B., et al. (2023). Cognitive Sparing in Proton versus Photon Radiotherapy for Pediatric Brain Tumor Is Associated with White Matter Integrity: An Exploratory Study. Cancers *15*, 1844. https://doi.org/10.3390/cancers15061844.

Michetti, C., Falace, A., Benfenati, F., and Fassio, A. (2022). Synaptic genes and neurodevelopmental disorders: From molecular mechanisms to developmental strategies of behavioral testing. Neurobiol. Dis. *173*, 105856. https://doi.org/10.1016/j.nbd.2022.105856.

Mohan, R., and Grosshans, D. (2017). Proton therapy – Present and future. Adv. Drug Deliv. Rev. *109*, 26–44. https://doi.org/10.1016/j.addr.2016.11.006.

Newman, A.M., Steen, C.B., Liu, C.L., Gentles, A.J., Chaudhuri, A.A., Scherer, F., Khodadoust, M.S., Esfahani, M.S., Luca, B.A., Steiner, D., et al. (2019). Determining cell type abundance and expression from bulk tissues with digital cytometry. Nat. Biotechnol. *37*, 773–782. https://doi.org/10.1038/s41587-019-0114-2.

Padovani, L., André, N., Constine, L.S., and Muracciole, X. (2012). Neurocognitive function after radiotherapy for paediatric brain tumours. Nat. Rev. Neurol. *8*, 578–588. https://doi.org/10.1038/nrneurol.2012.182.

Paganetti, H., Niemierko, A., Ancukiewicz, M., Gerweck, L.E., Goitein, M., Loeffler, J.S., and Suit, H.D. (2002). Relative biological effectiveness (RBE) values for proton beam therapy. Int. J. Radiat. Oncol. Biol. Phys. *53*, 407–421. https://doi.org/10.1016/S0360-3016(02)02754-2.

Parihar, V.K., and Limoli, C.L. (2013). Cranial irradiation compromises neuronal architecture in the hippocampus. Proc. Natl. Acad. Sci. USA *110*, 12822–12827. https://doi.org/10.1073/pnas.1307301110.

Parihar, V.K., Pasha, J., Tran, K.K., Craver, B.M., Acharya, M.M., and Limoli, C.L. (2015). Persistent changes in neuronal structure and synaptic plasticity caused by proton irradiation. Brain Struct. Funct. *220*, 1161–1171. https://doi.org/10.1007/s00429-014-0709-9.

Sabogal-Guaqueta, A.M., Mitchell-Garcia, T., Hunneman, J., Voshart, D., Thiruvalluvan, A., Foijer, F., Kruyt, F., Trombetta-Lima, M., Eggen, B.J.L., Boddeke, E., et al. (2024). Brain organoid models for studying the function of iPSC-derived microglia in neurodegeneration and brain tumours. Neurobiol. Dis. *203*, 106742. https://doi.org/10.1016/j.nbd.2024.106742.

Samarasinghe, R.A., Miranda, O.A., Buth, J.E., Mitchell, S., Ferando, I., Watanabe, M., Allison, T.F., Kurdian, A., Fotion, N.N., Gandal, M.J., et al. (2021). Identification of neural oscillations and epileptiform changes in human brain organoids. Nat. Neurosci. *24*, 1488–1500. https://doi.org/10.1038/s41593-021-00906-5.

Sebastian, R., Jin, K., Pavon, N., Bansal, R., Potter, A., Song, Y., Babu, J., Gabriel, R., Sun, Y., Aronow, B., and Pak, C. (2023). Schizophrenia-associated NRXN1 deletions induce developmental-timing- and cell-type-specific vulnerabilities in human brain organoids. Nat. Commun. *14*, 3770. https://doi.org/10.1038/s41467-023-39420-6.

Siegel, R.L., Miller, K.D., Wagle, N.S., and Jemal, A. (2023). Cancer statistics, 2023. CA Cancer J. Clin. *73*, 17–48. https://doi.org/10.3322/caac.21763.

Sienna, J., Kahalley, L.S., Mabbott, D., Grosshans, D., Santiago, A.T., Paulino, A.D.C., Merchant, T.E., Manzar, G.S., Dama, H., Hodgson, D.C., et al. (2024). Proton Therapy Mediates Dose Reductions to Brain Structures Associated With Cognition in Children With Medulloblastoma. Int. J. Radiat. Oncol. Biol. Phys. *119*, 200–207. https://doi.org/10.1016/j.ijrobp.2023.11.035.

Simó, M., Rodríguez-Fornells, A., Navarro, V., Navarro-Martín, A., Nadal, E., and Bruna, J. (2024). Mitigating radiation-induced cognitive toxicity in brain metastases: More questions than answers. Neurooncol Adv *6*, vdae137. https://doi.org/10.1093/noajnl/vdae137.

Sloan, S.A., Andersen, J., Paşca, A.M., Birey, F., and Paşca, S.P. (2018). Generation and assembly of human brain region–specific three-dimensional cultures. Nat. Protoc. *13*, 2062–2085. https://doi.org/10.1038/s41596-018-0032-7.

Sørensen, B.S., Pawelke, J., Bauer, J., Burnet, N.G., Dasu, A., Høyer, M., Karger, C.P., Krause, M., Schwarz, M., Underwood, T.S.A., et al. (2021). Does the uncertainty in relative biological effectiveness affect patient treatment in proton therapy? Radiother. Oncol. *163*, 177–184. https://doi.org/10.1016/j.radonc.2021.08.016.

Taylor, K.R., Barron, T., Hui, A., Spitzer, A., Yalçin, B., Ivec, A.E., Geraghty, A.C., Hartmann, G.G., Arzt, M., Gillespie, S.M., et al. (2023). Glioma synapses recruit mechanisms of adaptive plasticity. Nature *623*, 366–374. https://doi.org/10.1038/s41586-023-06678-1.

Trujillo, C.A., Gao, R., Negraes, P.D., Gu, J., Buchanan, J., Preissl, S., Wang, A., Wu, W., Haddad, G.G., Chaim, I.A., et al. (2019). Complex Oscillatory Waves Emerging from Cortical Organoids Model Early Human Brain Network Development. Cell Stem Cell *25*, 558–569.e7. https://doi.org/10.1016/j.stem.2019.08.002.

Turnquist, C., Harris, B.T., and Harris, C.C. (2020). Radiation-induced brain injury: Current concepts and therapeutic strategies targeting neuroinflammation. Neurooncol. Adv. *2*, vdaa057. https://doi.org/10.1093/noajnl/vdaa057.

Velasco, S., Kedaigle, A.J., Simmons, S.K., Nash, A., Rocha, M., Quadrato, G., Paulsen, B., Nguyen, L., Adiconis, X., Regev, A., et al. (2019). Individual brain organoids reproducibly form cell diversity of the human cerebral cortex. Nature *570*, 523–527. https://doi.org/10.1038/s41586-019-1289-x.

Voshart, D.C., Klaver, M., Jiang, Y., van Weering, H.R.J., van Buuren-Broek, F., van der Linden, G.P., Cinat, D., Kiewiet, H.H., Malimban, J., Vazquez-Matias, D.A., et al. (2024). Proton therapy induces a local microglial neuroimmune response. Radiother. Oncol. *193*, 110117. https://doi.org/10.1016/j.radonc.2024.110117.

Wang, X., Christian, K.M., Song, H., and Ming, G.L. (2018). Synaptic dysfunction in complex psychiatric disorders: from genetics to mechanisms. Genome Med. *10*, 9. https://doi.org/10.1186/s13073-018-0518-5.

Winter, S.F., Gardner, M.M., Karschnia, P., Vaios, E.J., Grassberger, C., Bussière, M.R., Nikolic, K., Pongpitakmetha, T., Ehret, F., Kaul, D., et al. (2024). Unique brain injury patterns after proton vs photon radiotherapy for whom grade 2-3 gliomas. Oncologist *29*, e1748–e1761. https://doi.org/10.1093/oncolo/oyae195.

Wu, M.-Y., Zou, W.-J., Yu, P., Yang, Y., Li, S.-J., Liu, Q., Xie, J., Chen, S.-Q., Lin, W.-J., and Tang, Y. (2022). Cranial irradiation impairs intrinsic excitability and synaptic plasticity of hippocampal CA1 pyramidal neurons with implications for cognitive function. Neural Regen. Res. *17*, 2253–2259. https://doi.org/10.4103/1673-5374.336875.

Zhang, D., Zhou, W., Lam, T.T., Weng, C., Bronk, L., Ma, D., Wang, Q., Duman, J.G., Dougherty, P.M., and Grosshans, D.R. (2018). Radiation induces age-dependent deficits in cortical synaptic plasticity. Neuro Oncol. *20*, 1207–1214. https://doi.org/10.1093/neuonc/noy052.

Zhang, W., Jiang, J., Xu, Z., Yan, H., Tang, B., Liu, C., Chen, C., and Meng, Q. (2023). Microglia-containing human brain organoids for the study of brain development and pathology. Mol. Psychiatry *28*, 96–107. https://doi.org/10.1038/s41380-022-01892-1.

Zhou, S., Ding, X., Zhang, Y., Liu, Y., Wang, X., Guo, Y., Zhang, J., Liu, X., Gong, G., Su, Y., et al. (2024). Evaluation of specific RBE in different cells of hippocampus under high-dose proton irradiation in rats. Sci. Rep. *14*, 8193. https://doi.org/10.1038/s41598-024-58831-z.

Stem Cell Reports, Volume *21*

Supplemental Information

Differential synaptic signaling responses in human cortical organoids after photon and proton irradiation

Yuting Jiang, Danieli Born Guerra, Daniëlle C. Voshart, Eline Hageman, Luiza Reali Nazario, Marc-Jan van Goethem, Rob P. Coppes, and Lara Barazzuol

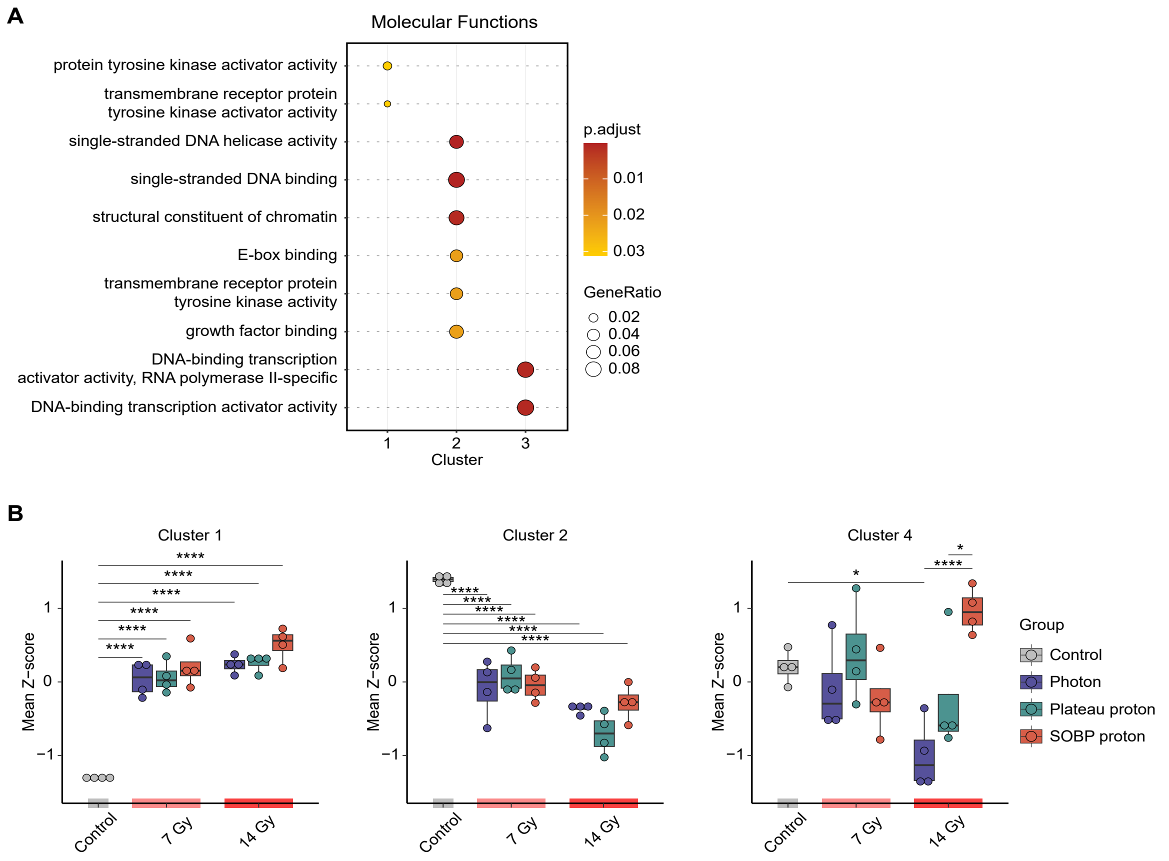

Figure S1. RNA-sequencing of hCOs after photon, plateau proton, and SOBP proton irradiation, related to Figure 1.

(A) Dot plot showing the top six enriched GO terms for molecular function in each cluster. (B) Boxplots showing the mean Z-score of genes in cluster 1, 2, and 4 for each condition. Boxes are drawn from first quartile to third quartile, with horizontal lines indicating the median. n = 4 pools per group, each pool contains 3-4 organoids. *p < 0.05 and ****p < 0.0001. Two-way ANOVA followed by Tukey's multiple comparisons test was used for data shown in (B).

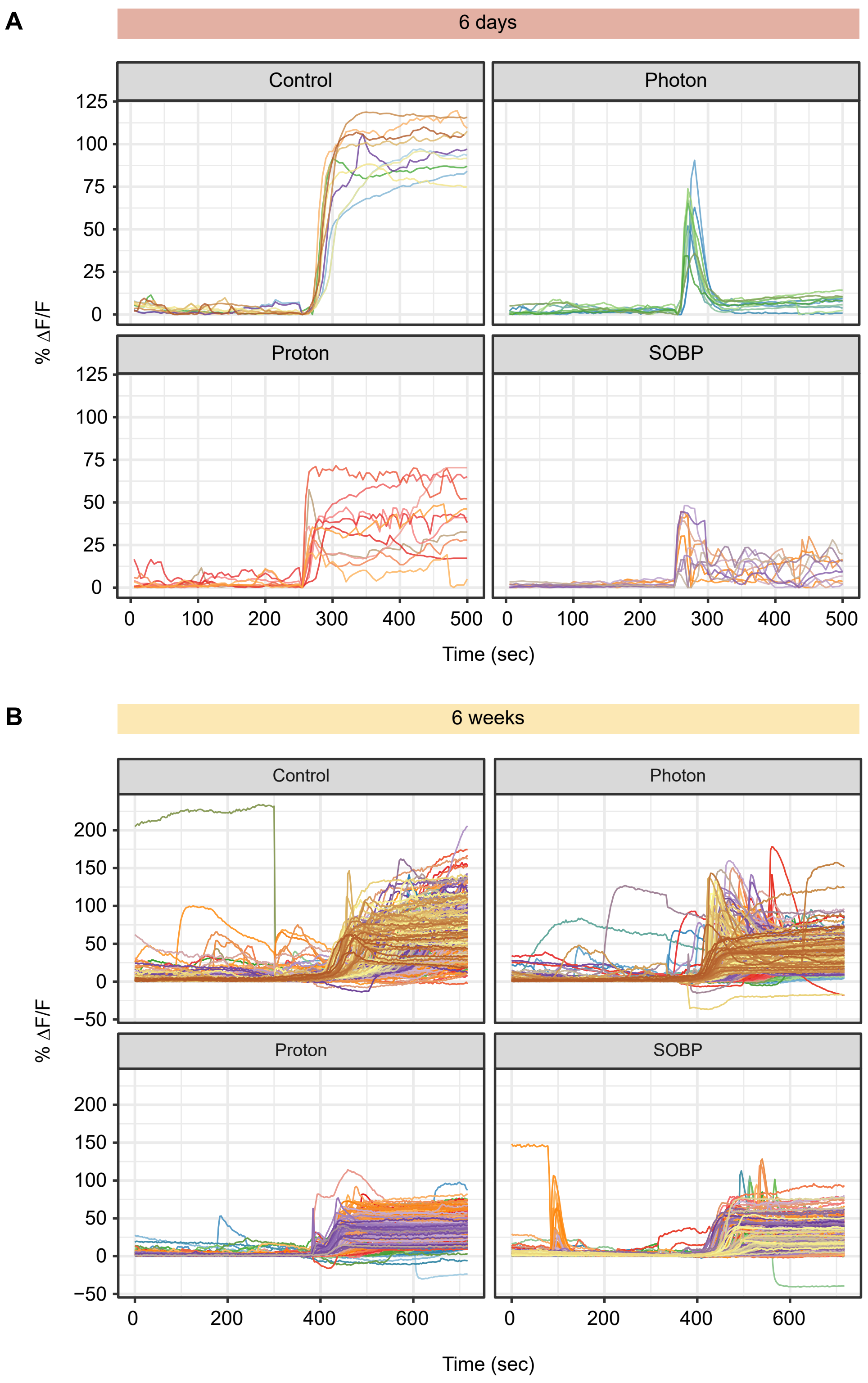

Figure S2. Individual spontaneous and glutamate-induced calcium traces at different time points after irradiation, related to Figure 2.

(A) Calcium traces from 10 cells per group at 6 days post-irradiation, with each line representing one cell. (B) Calcium traces from 300 cells per group at 6 weeks post-irradiation, with each line representing one cell.

Supplemental Methods

hCOs generation

HUES 9 (obtained from the Harvard Stem Cell Core Facility) hESCs were cultured on Matrigel (Corning, 734-1100) coated cell culture dishes using mTeSR1 medium (Stemcell Technologies, 85850). When HUES 9 cells were ready to be passaged, they were detached and dissociated into single cells using Accutase (Stemcell Technologies, 07920). The resulting cell suspension was counted and diluted in mTeSR1 medium containing ROCK inhibitor Y-27632 (1:100, Abcam, ab120129) before 3 million cells per well were seeded into AggreWell-800 plates (Stemcell Technologies, 34815). The plates were centrifuged at 100 g for 3 min to facilitate cell aggregation at the bottom of the microwells, which promotes uniform embryonic bodies (EB) formation. After 24h of incubation, EBs were transferred to ultra-low attachment T75 flasks (Merck, CLS3814) and cultured in neural induction medium (NIM), consisting of DMFM/F12 (Gibco, 11320-074), 20% knockout serum replacement (Gibco, 10828010), non-essential amino acids (1:100, Gibco, 11140050), Glutamax (Thermo Scientific, 35050038), 0.1 mM β-mercaptoethanol (Sigma, M3148), 1% penicillin-streptomycin (Gibco, 15140163), and supplemented with 5 μM dorsomorphin (Sigma, P5499-5MG) and 10 μM SB-431542 (Tocris, 1614). From day 6 onward, NIM was replaced daily with neural differentiation medium (NM) containing Neurobasal-A (Gibco, 10888022), 2% B-27 supplement without vitamin A (Gibco, 12587010), Glutamax (Thermo Scientific, 35050038), 1% penicillin-streptomycin (Gibco, 15140163), and supplemented with 20 ng/mL EGF (Sigma, E9644) and 20 ng/mL FGF-2 (PeproTech, 100-18B). Beginning on day 25, EGF and FGF-2 in NM were replaced by 20 ng/mL NT-3 (PeproTech, 450-03) and 20 ng/mL BDNF (PeproTech, 450-02). After day 43, the organoids were maintained in NM without any additional factors.

Animal irradiation

Adult male Wistar (Hsd/Cpb:WU) rats were kept under environmentally controlled conditions (temperature: 21°C, humidity: 55%) with a 12-hour light/dark cycle and ad libitum access to chow and water. The animals were housed in groups in open cages. All procedures were performed at the UMCG Central Animal Facility according to the guidelines from Directive 2010/63/EU of the European Parliament on the protection of animals used for scientific purposes. The experiments were approved by the Central Authority for Scientific Procedures on Animals (CCD) (license # AVD1050020184808) and the Animal Care and Use Committee of the University of Groningen. Rats were irradiated with 14 Gy photons or protons. Photon irradiations were conducted at the Central Animal Facility using an X-RAD 320 system (Precision X-Ray Inc., 200 kV, 1.559 Gy/min) with a specialized collimator for precise whole-brain targeting, as previously described (Voshart et al., 2024). Proton irradiations were performed at the Particle Therapy Research Center (PARTREC) accelerator facility, using either plateau protons (scattered beam, 150 MeV shoot-through, 15 Gy/min) or SOBP protons (4 Gy/min). Anesthesia was induced with 5% isoflurane and maintained at 1.5–2% during irradiation. Animals were sacrificed at 12 weeks post-irradiation by saline perfusion under dexmedetomidine-ketamine anesthesia. The brains were isolated and divided into two hemispheres, with one hemisphere fixed in 4% paraformaldehyde for 48 hours and subsequently embedded in paraffin for staining.

RNA isolation and RNA sequencing library preparation

RNA was isolated from hCOs 48 hours after irradiation using the RNeasy Lipid Tissue Mini Kit (Qiagen, 74804) following the manufacturer's instruction. Libraries were generated using the Lexogen QuantSeq 3' mRNA-seq Library Prep Kit (Lexogen, 015.96), with 200 ng of RNA used as input for each sample. All libraries were pooled in equimolar concentrations, and a 1.8 pM superpool with a 15% PhiX spike-in was sequenced on a NextSeq 500 using 75 bp single reads.

RNA sequencing data analysis

FASTQ files were aligned to the human genome (GRCh38) and pre-processed using the Quantseq Data Analysis Pipelines on the BlueBee Genomics Platform. Gene count matrices were used for the downstream analysis in R (v4.3.2). Genes with counts per million (CPM) greater than 2 in at least two samples were retained and normalized for differential gene expression analysis using edgeR (v4.0.14), with a threshold of 1.5 absolute fold change and an adjusted p-value < 0.05. GO analysis was conducted with clusterProfiler (v4.10.0) using the enrichGO function with a p-value and q-value cutoff of 0.05.

The CPM normalized gene expression matrix from this study was analyzed using the Docker version of CIBERSORTx to infer cell type compositions. Human single-cell brain organoid RNA-seq data containing control cells and cluster annotations were downloaded from

https://zenodo.org/record/7083558 (Li et al., 2023) and used to generate a custom signature matrix with the following arguments: --single_cell TRUE --replicates 200 --fraction 0.5 --verbose TRUE. Cell type fractions were then estimated using the following parameters: --perm 100 --rmbatchSmode TRUE --absolute FALSE.

Calcium imaging

Calcium activity in hCOs was visualized 6 days and 6 weeks after irradiation using the Fluo-4 Direct Calcium Kit (Invitrogen, F10471). Intact hCOs were placed in a 35 mm glass bottom plate containing NM without growth factors. Equal volumes of 2x Fluo-4 Direct calcium assay reagent were added to the medium. The hCOs were incubated at 37°C for 30 minutes, followed by 30 minutes at room temperature. The organoids were then transferred to the center of a crystal Petri dish for imaging. Fluorescence was recorded using a Leica DMI 8 microscope with a 40x objective for organoids at 6 days post-irradiation and a 10x objective for 6 weeks post-irradiation. Imaging was performed with an excitation wavelength of 494 nm and emission wavelength of 516 nm, capturing frames every 5 seconds for organoids at 6 days post-irradiation and every 3 seconds for 6 weeks post-irradiation. Basal calcium activity was recorded over 15 minutes. Stimulation experiments were performed by adding 4 µL of a 100 mM Glutamate stock solution directly to the Petri dish containing the organoid, and calcium responses were recorded for an additional 15 minutes.

Calcium imaging data were processed using ImageJ. Regions of interest (ROIs) representing individual cells were manually selected for organoids at 6 days post-irradiation and automatically segmented with the StarDist ImageJ plugin for organoids at 6 weeks post-irradiation. Mean gray values were measured for each frame. A total number of 10 cells were analyzed per condition at 6 days post-irradiation, and 300 cells were analyzed at 6 weeks post-irradiation. Relative changes in fluorescence (ΔF/F(t)) were calculated using the formula:

$$\frac{\Delta F}{F(t)} = \frac{F_t - F_0}{F_0}$$

where ΔF would be the difference of fluorescence intensities between the current frame and F_0, with F_0 represents minimum gray values of the time series of each ROI. Calcium transients were identified when ΔF/F(t) exceeded a threshold of 2 median absolute deviations. The peak amplitude was measured from the median line to the maximum value of the peak. For calcium transient frequency and amplitude analysis, 10 cells per condition were analyzed at 6 days post-irradiation, and 20 randomly selected cells out of 300 per condition were analyzed at 6 weeks post-irradiation. Cells that did not respond to glutamate within the analyzed time frame were excluded, resulting in 14-19 cells quantified per group at 6 weeks post-irradiation.

Immunofluorescence staining

hCOs were collected 6 days post-irradiation and fixed an hour at room temperature in 4% PFA. After fixation, the organoids were transferred to 30% sucrose at 4 °C until they sank, and then embedded in OCT compound. Sections of 10 µm thickness were cut using a Leica Cryostat (Leica, CM1860). Cryosections were allowed to re-equilibrate at room temperature for 30 minutes, followed by a 10 minute wash in PBS to remove OCT. Antigen retrieval was performed using 10 mM sodium citrate buffer with 0.05% Tween-20 (pH 6.0). Sections were then blocked for 1 hour at room temperature in blocking solution containing 4% goat or donkey serum depending on the species of secondary antibodies, 1% BSA, and 0.1% Triton X-100 in PBS, and incubated overnight at 4 °C with the following primary antibodies diluted in blocking solution: chicken anti-Homer1 (Synaptic Systems, 160 006, 1:500, RRID: AB_2631222), guinea pig anti-VGLUT1 (Synaptic Systems, 135 304, 1:2000, RRID: AB_887878), rabbit anti-TUBB3 (Sigma, T2200, 1:500, RRID: AB_262133), rat anti-CTIP2 (Abcam, ab18465, 1:250, RRID: AB_2064130), rabbit anti-PAX6 (BioLegend, 901301, 1:100, RRID: AB_2565003). After primary antibody incubation, sections were washed and incubated for 1 hour at room temperature with appropriate secondary antibodies diluted in blocking solution: Alexa Fluor 488 goat anti-chicken (Invitrogen, A-11039, 1:500, RRID: AB_2534096), Alexa Fluor 594 goat anti-guinea pig (Invitrogen, A-11076, 1:500, RRID: AB_2534120), Alexa Fluor 647 goat anti-rabbit (Invitrogen, A-21245, 1:500, RRID: AB_2535813), Alexa Fluor 488 donkey anti-rabbit (Invitrogen, A-21206, 1:500, RRID: AB_2535792), Alexa Fluor 594 donkey anti-rat (Invitrogen, A-21209, 1:500, RRID: AB_2535795). Nuclei were counterstained with DAPI.

For immunofluorescence staining of rat brain tissue, 5 µm-thick paraffin sections were deparaffinized, rehydrated, and subjected to antigen retrieval by boiling in 10 mM sodium citrate buffer with 0.05%

Tween-20 (pH 6.0) for 10 min. The remaining staining procedure was performed as described above for the organoid sections.

Confocal microscopy and image analysis

To evaluate the density of excitatory synapses, images were acquired using a Leica SP8X confocal microscope with 60x magnification, 4x digital zoom, 0.3 μm z-step size, and Lightning mode for deconvolution. At least four random regions per organoid and three to four regions per rat brain sample were imaged. Pre- and post-synaptic puncta were identified using the “Spots” identifier in Imaris software (Bitplane, v9.7.2) with background subtraction enabled. The average diameter of randomly selected puncta was measured and applied for spot detection. Detection parameters were set to an xy diameter of 0.5 μm and z diameter of 1 μm for pre-synaptic puncta and an xy diameter of 0.4 μm and z diameter of 0.8 μm for post-synaptic puncta. Artifact spots not representing synaptic puncta were excluded by adjusting the “Quality” and “Intensity StdDev” thresholds. The “Colocalize Spots” function was used to identify adjacent pre- and post- synaptic puncta within a distance of 0.5 μm. Neuronal processes, delineated using the “Surfaces” function based on TUBB3 staining, were used to normalize synaptic density measurements.

Cell densities in VZ were quantified by acquiring images around the VZ using a Leica SP8X confocal microscope with 40x magnification. Image analysis was performed with ImageJ. Nuclei segmentation was performed using the StarDist ImageJ plugin on DAPI channel with default settings. A ROI encompassing the VZ was defined in each image based on cell orientation and the density of DAPI^{+}, PAX6^{+} and CTIP2^{+} cells. Only nuclei within the ROI were included for intensity measurements in PAX6 and CTIP2 channels. Cells were classified as PAX6^{+} and CTIP2^{+} if their mean fluorescence intensity above a specific threshold. Threshold values were adjusted to optimize the detection of visually identifiable PAX6^{+} and CTIP2^{+} cells while minimizing false positives.

Supplemental references

Li, C., Fleck, J. S., Martins-Costa, C., Burkard, T. R., Themann, J., Stuempflen, M., Peer, A. M., Vertesy, Á., Littleboy, J. B., Esk, C., Elling, U., Kasprian, G., Corsini, N. S., Treutlein, B., & Knoblich, J. A. (2023). Single-cell brain organoid screening identifies developmental defects in autism. *Nature*, *621*(7978), 373–380. https://doi.org/10.1038/s41586-023-06473-y

Voshart, D. C., Klaver, M., Jiang, Y., van Weering, H. R. J., van Buuren-Broek, F., van der Linden, G. P., Cinat, D., Kiewiet, H. H., Malimban, J., Vazquez-Matias, D. A., Reali Nazario, L., Scholma, A. C., Sewdihal, J., van Goethem, M.-J., van Luijk, P., Coppes, R. P., & Barazzuol, L. (2024). Proton therapy induces a local microglial neuroimmune response. *Radiotherapy and Oncology*, *193*(Accepted), 110117. https://doi.org/10.1016/j.radonc.2024.110117
